# Review on the Degradation of Poly(lactic acid) during Melt Processing

**DOI:** 10.3390/polym15092047

**Published:** 2023-04-25

**Authors:** Ineke Velghe, Bart Buffel, Veerle Vandeginste, Wim Thielemans, Frederik Desplentere

**Affiliations:** 1 Processing of Polymers and Innovative Material Systems ProPoliS, Department of Materials Engineering, KU Leuven Campus Bruges, Spoorwegstraat 12, 8200 Bruges, Belgium; bart.buffel@kuleuven.be; 2 Surface and Interface Engineering Materials, Department of Materials Engineering, KU Leuven Campus Bruges, Spoorwegstraat 12, 8200 Bruges, Belgium; veerle.vandeginste@kuleuven.be; 3 Sustainable Materials Lab, Department of Chemical Engineering, KU Leuven Campus Kulak Kortrijk, Etienne Sabbelaan 53, 8500 Kortrijk, Belgium; wim.thielemans@kuleuven.be

**Keywords:** poly(lactic acid), PLA, degradation, melt processing

## Abstract

This review paper presents an overview of the state of the art on process-induced degradation of poly(lactic acid) (PLA) and the relative importance of different processing variables. The sensitivity of PLA to degradation, especially during melt processing, is considered a significant challenge as it may result in deterioration of its properties. The focus of this review is on degradation during melt processing techniques such as injection molding and extrusion, and therefore it does not deal with biodegradation. Firstly, the general processing and fundamental variables that determine the degradation are discussed. Secondly, the material properties (for example rheological, thermal, and mechanical) are presented that can be used to monitor and quantify the degradation. Thirdly, the effects of different processing variables on the extent of degradation are reviewed. Fourthly, additives are discussed for melt stabilization of PLA. Although current literature reports the degradation reactions and clearly indicates the effect of degradation on PLA’s properties, there are still knowledge gaps in how to select and predict the processing conditions that minimize process-induced degradation to save raw materials and time during production.

## 1. Introduction

Plastics play an essential role in today’s society, since they are low-cost, lightweight, versatile, and easily processable materials that possess excellent mechanical, barrier, and aesthetic properties [1,2,3]. Consequently, the large-scale production of petroleum-based plastics has since the 1950s [4] resulted in their use in an enormous variety of market segments, such as packaging, healthcare, automotive, clothing, etc. As a result of society’s mass consumption and because plastics continue to replace other conventional materials such as glass or metal, the exponential growth in plastic production is expected to continue through the coming decades [5]. The worldwide production of 367 million tons of plastic in 2021 [6] is predicted to increase to 600 million tons of plastic in 2025 [7].

Because most plastics are based on non-renewable resources such as crude oil or natural gas, the increasing demand has resulted in both environmental and economic consequences: the massive accumulation of mainly non-degradable plastic waste in nature [8], the contributions to climate change and air pollution by toxic emissions and carbon dioxide generated during the incineration of plastic waste [9], the expected unstable and increasing oil and gas prices due to the depletion of fossil raw materials [10], and the pollution of the food chain by fragmented plastic particles that are mostly non-degradable [11]. Worldwide climate and environmental concerns have resulted in industrial efforts to develop bioplastics, to lessen society’s dependency on non-renewable resources, and to limit the enormous pile of plastic waste in the environment [12,13].

Bioplastics can be biobased, biodegradable, or a combination of both [14]. Biobased plastics are plastics obtained from natural, renewable resources such as cellulose or corn starch [15], while biodegradable polymers degrade into water and carbon dioxide through microbial action from bacteria, algae, and fungi. The increasing interest in bioplastics has resulted in a large variety of commercially available materials, that are promising candidates to replace petroleum-based plastics. The most widely studied bioplastics are the aliphatic polyesters, such as poly(lactic acid) (PLA), polyhydroxyalkanoates (PHAs), bio-polybutylene succinate (PBS), and polybutylene adipate terephthalate (PBAT), which can either be petroleum- or bio-based. Since the ester groups of polyesters are susceptible to hydrolysis in the presence of moisture, they benefit from being biodegradable materials [3].

In 2022, global bioplastic-production consisted of 20.7% of PLA [16], making it the most extensively studied and used bioplastic worldwide. Several beneficial properties of PLA contribute to why the market demand is expected to further increase in the coming years [5]. PLA is a thermoplastic polyester that is both biodegradable and biobased (since it can be derived from corn starch and sugarcane) [17], it can be processed using traditional polymer processing equipment (such as injection molding (IM) and extrusion) [18], and it is commercially available at large scale [19]. PLA has comparable optical, barrier, mechanical, and thermal properties to petroleum-based polymers such as polystyrene (PS) and polyethylene terephthalate (PET); therefore, it can serve as a sustainable alternative to these conventional plastics [20]. The environmental impact of PLA is considered low in comparison with PET, with a life cycle assessment (LCA) showing that PLA production and waste disposal results in a reduction of greenhouse gas emissions, global warming impact, human toxicity, and fossil energy consumption [21].

**Figure 1:**
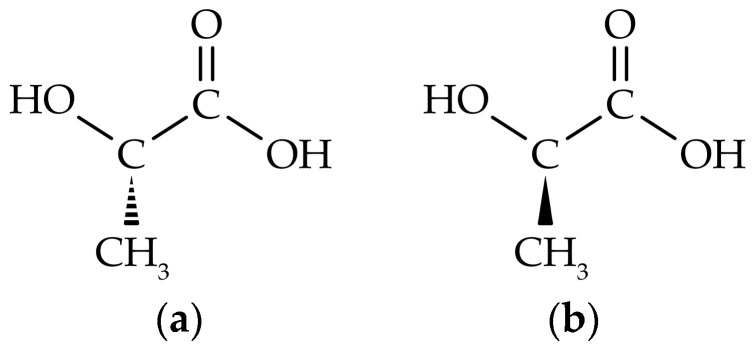
Chemical structure of (**a**) L-lactic acid and (**b**) D-lactic acid.

The polymer backbone of PLA is produced from the monomer lactic acid, a fermentation product of sugar feedstock that occurs in two optically active enantiomers, namely L-lactic acid and D-lactic acid [22], illustrated in Figure 1. There are three main ways to produce PLA: (1) direct condensation polymerization, (2) direct polycondensation in an azeotropic solution, and (3) ring-opening polymerization (ROP) from lactide, a cyclic dimer of lactic acid. ROP is industrially the preferred way to produce PLA, since it is able to result in high molecular weight PLA with a higher commercial value [13,20,23]. A large spectrum of commercial grades is produced, varying in molecular weight (Mw), and in the ratio of L- to D-lactic acid. Since the majority of lactic acid in nature occurs as the L-enantiomer, commercial polymer chains will consist mainly of L-lactic acid [1], unless specific care is taken to synthesize polymers with a high amount of D-lactic acid. The percentage of D-enantiomer will define whether the polymer can crystallize or not. A highly semi-crystalline material is obtained for a D-isomer content below 1.5% [24], while amorphous PLA is obtained for a D-isomer content above 7% [23]. Since the degree of crystallinity ($X_{c}$) affects the glass transition temperature ($T_{g}$), the melting temperature ($T_{m}$), and the mechanical properties [25], a large spectrum of PLA grades is available to match different technical requirements for biomedical, agricultural, food packaging, textile, and 3D printing applications [23,26].

The same ester groups in aliphatic polyesters that are responsible for biodegradation, are vulnerable linkages during melt processing [27]. Therefore, the main issue in processing PLA is its extreme sensitivity to high temperature, moisture, and shear [28]. A narrow processing window for PLA is the result of a trade-off between processing the material under the mildest conditions possible to minimize degradation, and also obtaining good homogeneity and optimal melt processing requirements (pressure and flow rate) [29]. Polymer chains undergo thermal, thermomechanical, and hydrolytic degradation due to high temperature and shear, a long residence time inside the equipment, and a high moisture content, which results in a significant loss in molecular weight [22,30]. It is crucial to understand the influence of processing conditions on this molecular weight decrease, since it results in a decrease in the quality of the end product due to deterioration of the thermal, rheological, and mechanical properties [31], and it causes damage to the equipment by volatile lactide that is released [32]. Suppressing the degradation in the melt by adjusting the processing parameters has advantages for industrial processors of PLA, since it will reduce the production cost, the amount of post-production waste, the energy losses, and the use of raw materials, to end up with high quality and reproducible polymer products [19,30,33]. This review article presents the current literature on the effects of different processing conditions on the degradation of PLA during melt processing. For a better understanding of the results in the literature discussed in this review, a brief summary of PLA degradation mechanisms and an introduction to polymer processing conditions is provided, together with different methods to measure and quantify PLA degradation. In addition, additives are discussed for melt stabilization of PLA. Biodegradation or degradation during use of PLA is not discussed here, since this review article focusses specifically on the effect of melt processing on its degradation.

## 2. Degradation Mechanisms during Melt Processing

### 2.1. Melt Processing

Thermoplastic polymers such as PLA need to be converted from simple granules into more complex consumer products by using well-established polymer processing technologies such as extrusion and injection molding [3]. Before PLA is melt-processed, the amorphous or semi-crystalline granules are dried to remove residual moisture since PLA is a hygroscopic material that absorbs moisture from the atmosphere. Therefore, the main supplier NatureWorks™ (Minnetonka, MN, USA) recommends drying PLA until the moisture content is below 250 ppm [34]. Multiple industrial processers even avoid processing PLA that contains a moisture content higher than 100 ppm, to prevent severe degradation as discussed in Section 2.2 [23].

**Figure 2:**
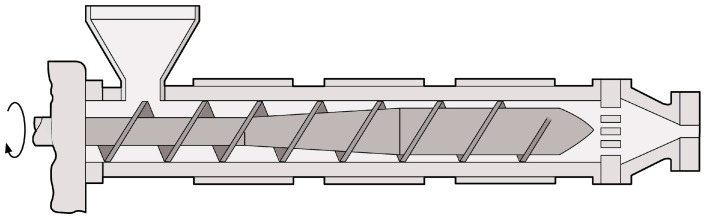
Working principle of the single-screw extrusion process.

**Figure 3:**
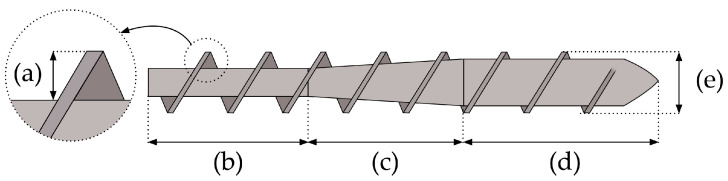
Geometry of a basic screw with (**a**) flight depth, (**b**) feed zone, (**c**) compression zone, (**d**) metering zone, and (**e**) outer diameter D.

The major technique for melting thermoplastic polymers is screw extrusion (Figure 2). A screw extruder consists of a hopper for feeding the granules, an electrically heated barrel, a screw, a motor for rotating the screw, and a die. The molten plastic is pushed through the die and cooled with air or in a cooling bath, resulting in products with a continuous cross-section like profiles or tubes. Melting of the PLA occurs through the combined heat generated by the heater bands and the shear heating created by the friction between the granules and the screw and barrel [3]. The simplest version of a single-screw extruder (SSE) is illustrated in Figure 3. It contains a screw that is characterized by a L/D ratio (ratio between the length L and the outer diameter D of the screw) and consists of three zones: (1) the feed zone, (2) the compression zone, and (3) the metering zone [35]. The ratio between the flight depth in the feed section and the flight depth in the metering section is called the compression ratio of the screw. The screw design is of great importance, since it will determine the residence time and shear exerted on the melt. Other important processing parameters are the temperature along the barrel, the selected die geometry, and the screw rotation speed [3,35,36].

**Figure 4:**
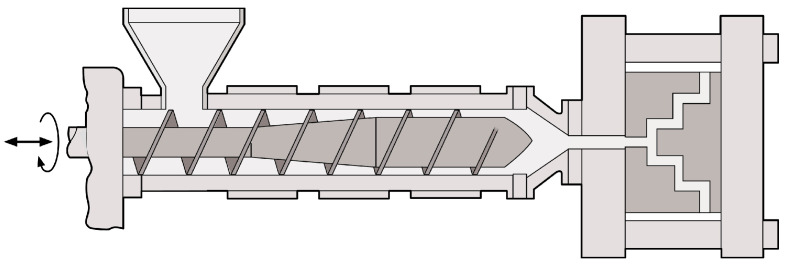
Working principle of the injection molding process.

Although screw extrusion is a continuous process, injection molding is the most used polymer processing technique to make individual, complex products (Figure 4). An injection molding machine also consists of a hopper, a heated barrel, a reciprocating screw (can both rotate and translate), and a mold equipped with a cooling system and ejector pins. The PLA is melted by the rotating screw and the heater bands, followed by injecting it into the mold cavity by the forward-moving screw. Finally, the polymer melt is cooled down and the solidified product is ejected from the mold. The injection molding process is characterized by a fast filling rate and high injection and holding pressures, resulting in high shear on the polymer molecules [3].

Extrusion and injection molding are the two main melt processing techniques used in industry. Many other processing techniques such as blow molding, cast extrusion, thermoforming, 3D-printing, and rotation molding are used in industry, but they are either derived from extrusion processes, or less widespread in industry and therefore considered to be out of scope for this review article.

A large variety of processing parameters can be changed to control and optimize the extrusion and injection molding process. Nevertheless, these can be reduced to four fundamental parameters: (1) moisture content in the polymer, (2) processing temperature, (3) residence time, and (4) shear. For example, the screw rotation speed will affect both the residence time and the shear of the melt during extrusion. Also, large shear (due to the die geometry or high compression ratio of the screw) will create shear heating, which affects the processing temperature. Altogether, these four fundamental parameters determine the amount of process-induced degradation of the polymer.

### 2.2. Degradation Mechanisms

Degradation of PLA describes any mechanism that results in shortening of the polymer chains and reduction in the molecular weight [37], caused by different factors such as heat, mechanical stress, oxygen, moisture, etc. [33,38,39]. Since melt processing is dominated by the above-mentioned four fundamental parameters (moisture, temperature, residence time, and shear), the total degradation of PLA during melt processing is a combination of thermal, hydrolytic, and thermomechanical degradation:

- Hydrolytic degradation is the cleavage of ester linkages in the polymer backbone when PLA is exposed to moisture, resulting in carboxyl and hydroxyl linear polymers or oligomers with shorter chain length [40]. The splitting of the ester group through hydrolysis is dependent on the water content and can occur during melt processing or during exposure to water. Both mechanisms of hydrolytic degradation result in a reduction in the molecular weight due to random chain scission of the polymer backbone [20,23,41].
- Thermal degradation occurs when PLA is exposed to high temperatures, becoming effective above 100 °C through the scission of the ester bonds [42]. A complex combination of six degradation reactions takes place: hydrolysis, intramolecular and intermolecular transesterification, homolysis, pyrolytic elimination, and radical degradation [23,39,43]. Hydrolysis (a) can be activated during melt processing when PLA is exposed to water at high temperature, as introduced before. The extent of the degradation depends on the residual moisture in the granules, which is why it is recommended to dry PLA before melt processing, as discussed in Section 2.1 [20,23,41]. Intramolecular transesterification (b), both at the end of the chain (backbiting) and in the middle of the chain, is the dominant degradation reaction during melt processing of PLA at temperatures above 200 °C. This reaction leads to the formation of cyclic and linear oligomers with a shorter chain length [44]. Intermolecular transesterification (c) is the exchange of ester units between different chains, resulting in a variation in the molecular weight distribution [40]. Homolysis (d), pyrolytic elimination (e), and radical degradation (f), are less dominant in thermal degradation [43]. These reactions are either categorized into chain scission processes (random degradation) or backbiting processes (chain-end degradation) [45]. Random degradation occurs at a random point along the polymer chain, resulting in the formation of shorter chain fragments, a decrease in molecular weight, and limited liberation of monomers (see Equation (1)). During chain-end degradation, also called unzipping depolymerization, the degradation occurs by backbiting processes at the end of the chain, causing the release of monomer units (see Equations (2) and (3)) and thereby a slower reduction of the molecular weight.

(1) $$M_{n}\to M_{x}+M_{y}\;(random\;degradation)$$

(2) $$M_{n}^{*}\to M_{n-1}^{*}+M\;(chain\;end\;degradation)$$

(3) $$M_{n-1}^{*}\to M_{n-2}^{*}+M\;(chain\;end\;degradation)$$

- Thermomechanical degradation occurs when PLA is exposed to high mechanical stresses due to elongational and shear flow during processing. Mechanical loading causes the breakage of the long polymer chains into shorter fragments, as well as shear heating [46,47]. Both terms, mechanical and thermomechanical degradation, are used in the literature, although the use of thermomechanical is preferred since mechanical loading causes an increase in temperature due to shear heating.

Depending on the selected processing characteristics, one of the above-described mechanisms will dominate PLA degradation. This emphasizes the importance of a proper selection of processing methods and conditions, since they can influence the extent of the process-induced degradation and minimize the undesired decrease in molecular weight.

## 3. Measuring Process-Induced Degradation

As a result of the degradation reactions, changes in chemical structure, molecular weight distribution, and mechanical, thermal, rheological, and permeability properties may occur. In order to identify the effect of processing on the properties, both in-line and off-line measurements can be adopted. Off-line measurements are used to investigate the properties of the extrudates after PLA is processed, whereas in-line measurements can identify degradation while PLA is melt-processed. This section will provide an overview of different off- and in-line measurements that can be used to monitor the extent of PLA-degradation.

### 3.1. Off-Line Measurements

To understand the effect of processing on the properties of the end-product, off-line measurements are crucial. Degradation of PLA can be quantified by investigating the molecular weight distribution and chemical structure after processing, and by studying the thermal, rheological, mechanical, and visual properties of the extrudates.

#### 3.1.1. Molecular Weight Distribution

The most obvious method to investigate process-induced degradation of PLA is studying the molecular weight distribution of virgin and processed PLA, since melt processing causes cleavage of the chains into shorter fractions. Gel permeation chromatography (GPC) is used to investigate the average number-molecular weight ($\overset{-}{M_{n}}$), the average weight-molecular weight ($\overset{-}{M_{w}}$), and the polydispersity index (Đ). Chávez-Montez et al. [48] reported a reduction of $\overset{-}{M_{n}}$ from 81.4 kg/mol for virgin PLA to 57.0 kg/mol after twin-screw extrusion, while $\overset{-}{M_{w}}$ decreased from 270.0 kg/mol to 157.8 kg/mol. Additionally, Meng et al. [38] reported a decrease in $\overset{-}{M_{n}}$ and $\overset{-}{M_{w}},$ from 92.6 kg/mol and 112 kg/mol to 83.8 kg/mol and 104 kg/mol, respectively. Other researchers [5,12,17,20,28,29,30,43,49,50,51,52,53,54,55,56,57,58,59] reported the same decreasing trend in molecular weight, which makes $\overset{-}{M_{n}}$ and $\overset{-}{M_{w}}$ good indicators to monitor process-induced degradation. Whether the decrease is small or large depends on the selected processing technique and parameters, as will be further discussed in Section 4.

**Figure 5:**
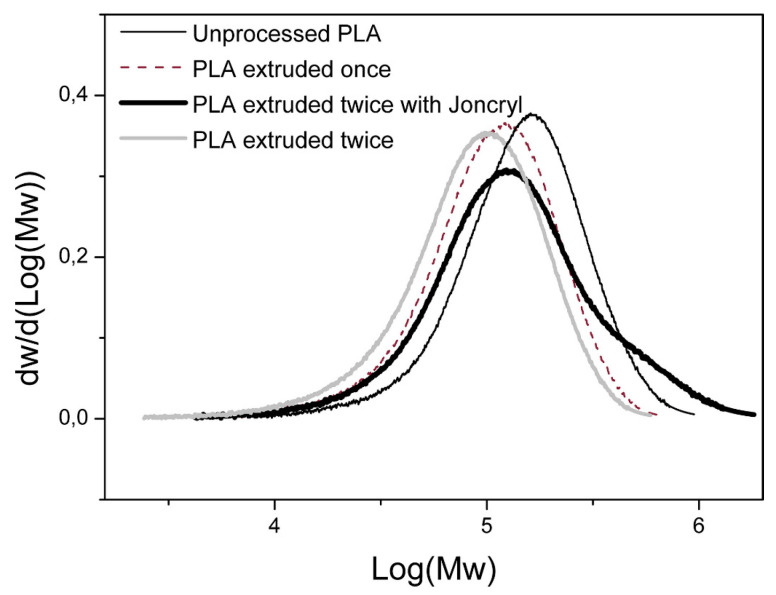
GPC curves for unprocessed PLA and PLA that was extruded once or twice. Reprinted with permission from Ref. [55]. 2023, John Wiley and Sons.

Also, the Đ ($\overset{-}{M_{w}}$/$\overset{-}{M_{n}}$) is reported in literature, which is a measure of the broadness of the molecular weight distribution. Some authors [5,30,48,50,51,53] concluded that the Đ remains stable, which can be explained by the random chain scission reactions that dominate degradation. Other authors, such as Cosate De Andrade et al. [55] and Gamez-Perez et al. [12], reported an increase in Đ after processing from 1.6 to 1.7 and from 2.82 to 3.03, respectively. The increase in Đ is explained by the formation of some shorter chains due to degradation, whereas other chains will remain long and thereby broaden the molecular weight distribution, as illustrated in Figure 5. Benvenuta-Tapia and Vivaldo-Lima [17], Jain et al. [54], and Tesfaye et al. [28] also reported an increase in the Đ due to degradation, but no clear explanation was given. In the work of Lv et al. [52], a Đ decrease was reported from 1.95 for unprocessed PLA to 1.83 after 4 reprocessing cycles, which is explained by the degradation occurring mainly in the chains with a high molecular weight. Amini Moghaddam et al. [49] also reported a decrease in Đ, but no explanation was given. In the work of Wang et al. [47], unprocessed PLA had a Đ of 1.98, whereas the Đ of extrudates varied between 1.63 and 2.50. Although a large variety in Đ was observed, it was concluded that there was no significant change in Đ due to degradation. Due to the contradictory findings, the Đ is a less straightforward indicator to monitor process-induced degradation.

The molecular weight can be used to quantify the extent of process-induced degradation. A degradation parameter *K* is used in literature, which is in general the ratio between the average number- or average weight-molecular weight of unprocessed PLA ($\overset{-}{Mn}{}_{unprocessed}$ or $\overset{-}{Mw}{}_{unprocessed}$) and the average number- or average weight-molecular weight of the processed materials ($\overset{-}{Mn}{}_{processed}$ or $\overset{-}{Mw}{}_{processed}$), as stated in Equation (4):

(4) $$K=\frac{\overset{-}{Mn}{}_{unprocessed}}{\overset{-}{Mn}{}_{processed}}\;or\;K=\frac{\overset{-}{Mw}{}_{unprocessed}}{\overset{-}{Mw}{}_{processed}}$$

Different interpretations of these equations are used in literature to calculate *K* to quantify the extent of the degradation. Signori et al. [39] and Al-Itry et al. [20] calculated *K* $\left(\overset{-}{Mn}{}_{pellets}/\overset{-}{Mn}{}_{T}\right)$ by comparing the average number-molecular weight of unprocessed pellets ($\overset{-}{Mn}{}_{pellets})$ and the average number-molecular weight after processing PLA at different temperatures between 150 °C and 200 °C ($\overset{-}{Mn}{}_{T}$), both concluding an increase in K with increasing processing temperature. Tesfaye et al. [28] and Cosate De Andrade et al. [55] calculated *K* $\left(\overset{-}{Mw}{}_{pellets}/\overset{-}{Mw}{}_{reprocessed}\right)$ by comparing the average weight-molecular weight of the unprocessed pellets ($\overset{-}{Mw}{}_{pellets}$) and the average weight-molecular weight of five or two reprocessing cycles PLA ($\overset{-}{Mw}{}_{reprocessed}$), respectively. Both studies reported an increase in *K* as the number of reprocessing cycles increased, indicating a decrease in molecular weight due to degradation.

#### 3.1.2. Rheological Properties

Rheological properties of polymers are crucial during polymer processing and they are directly linked to the molecular weight of the material. Thereby, different researchers use the viscosity of a polymer to monitor the degradation by performing different measurements: (1) complex viscosity (η*) and zero-shear viscosity (η_0_) with a parallel plate rheometer; (2) apparent viscosity (η_app_) with a capillary rheometer; and (3) intrinsic viscosity ([η]) linked to the viscous molar mass (M_v_).

**Figure 6:**
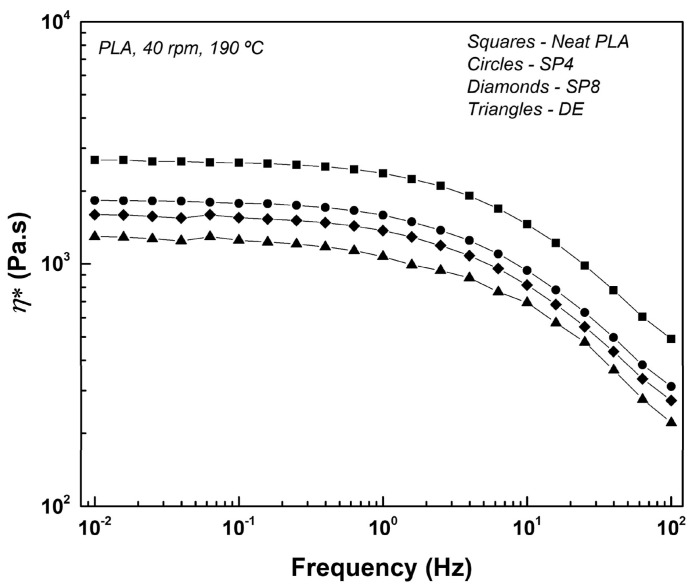
Rheological dynamic frequency sweep measurements for neat PLA and PLA samples collected at sampling port 4 (SP4), sampling port 8 (SP8), and at die exit (DE). Reprinted with permission from Ref. [60]. 2023, Elsevier.

A first method is to perform dynamic frequency sweep measurements with a parallel plate rheometer to investigate the process-induced degradation of PLA. An often-used indicator is η_0_, which is the constant melt viscosity of a polymer at low shear rates or frequencies, and it is proportional to the molecular weight ($\eta_{0}\sim M_{w}^{3.4}$) [60]. Benvenuta-Tapia and Vivaldo-Lima [17] reported a decrease of η_0_ from 2300 Pa·s for neat PLA to 330 Pa·s for processed PLA. Teixeira et al. [60] investigated the degradation during PLA extrusion, by taking samples at multiple locations along the extrusion screw, as illustrated in Figure 6. The frequency sweep measurements showed a decrease in η_0_ from around 2680 Pa·s of neat PLA before processing to around 1300 Pa·s at the die end, which indicates degradation of the PLA chains along the extrusion process. Brüster et al. [50], Botta et al. [61], and Scaffaro et al. [62] investigated the effects of multiple melt processing cycles on the degradation, all concluding a decrease of η* when the number of melt processing cycles increases. Other researchers [20,30,33,59,63,64] reported a similar decrease in η*. Also in the work of Tesfaye et al. [65], a decrease in η* was observed from 1730 Pa·s to 180 Pa·s after four reprocessing cycles, but also changes in storage modulus (G′), loss modulus (G″), and relaxation time were reported. Both G′ and G″ decreased when the number of reprocessing cycles increased, but also the characteristic relaxation time decreased from 0.006 s for neat PLA to <0.001 s after 4 reprocessing cycles. Cuadri and Martín-Alfonso [33] confirmed these findings by reporting a decrease in G′ and G” at 180 °C, 200 °C, and 220 °C in an air- or N_2_-atmosphere. The decreases in η*, G′, G″, and relaxation time are a direct result of the decrease in molecular weight, which, as previously stated, is a result of thermal, thermomechanical, and hydrolytic degradation mechanisms during processing. It can be concluded that η* and η_0_ are good indicators to monitor the degradation of PLA during processing, and that dynamic frequency sweeps are an alternative for GPC to estimate the decrease in molecular weight.

A second method is to use capillary rheometry to monitor the degradation of PLA, as reported by Nekhamanurak et al. [64] and Peinado et al. [66]. The η_app_ of extruded PLA was concluded to be lower than the η_app_ of neat PLA, also due to the decrease in molecular weight as a result of degradation processes. Although a capillary rheometer measures the viscosity at shear rates higher than 10 s^−1^ and makes it thereby impossible to measure η_0_, it can still be used to monitor process-induced degradation. It is important to emphasize that the thermomechanical loading during capillary rheometry is not identical to industrial processes such as extrusion or injection molding, since only conductive heating occurs and the melt is sheared shortly when it is being pushed through the capillary.

A third method is to perform intrinsic viscosity measurements, as in the work of Al-Itry et al. [20], where a decrease in [η] from 125 mL/g to 111 mL/g for melt-processed PLA was reported. Béltran et al. [58] reported a decrease from 132 mL/g to 109 mL/g during processing. This decrease in [η] is linked to a decrease in viscous molar mass, which was reported by Botta et al. [61]. In this work, a decrease in M_v_ of 22% was reported after five reprocessing cycles of PLA. Other researchers [18,38,65,67] found similar results, showing that [η] and M_v_ can be used to measure process-induced degradation of PLA.

**Figure 7:**
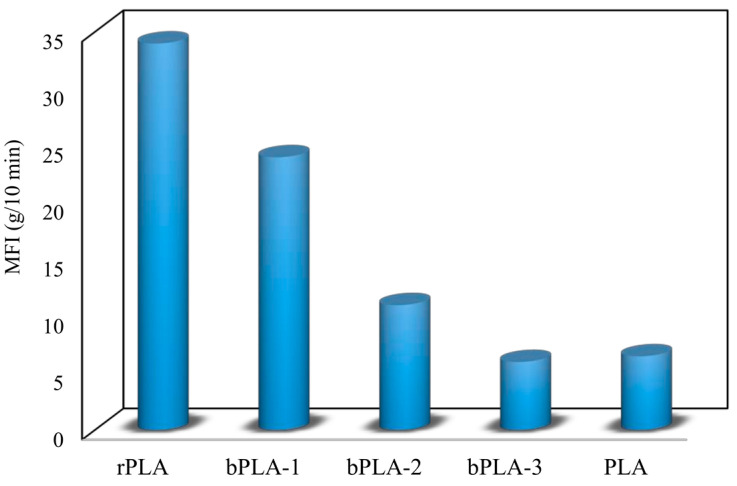
Melt flow index (MFI) for PLA, rPLA, and chain extended rPLA blends [70].

All the aforementioned methods to measure viscosity can be used to monitor degradation during processing of PLA. As an alternative for expensive viscosity equipment, the melt flow index (MFI) is often used in literature and industry because of fast and simple measurements. The MFI of a polymer is inversely proportional to its viscosity, and can be used to indirectly make assumptions about changes in viscosity and molecular weight due to degradation. All relevant studies [12,26,40,55,68,69,70] concluded an increase in MFI upon melt processing of PLA. In the work of Gamez-Perez et al. [12], an increase in MFI was reported, from 7.0 g/10 min for neat PLA to 7.4 g/10 min after twin-screw extrusion. Also, Benvenuta Tapia and Vivaldo-Lima [70] observed an increase from 6.5 g/10 min to 34 g/10 min after 2 subsequent extrusion cycles simulating the mechanical recycling process (see Figure 7). This increase in MFI can be easily explained: when the molecular weight and thus also the viscosity decreases because of degradation, the melt is able to flow faster and thereby a higher MFI is obtained. Rojas-Gonzalez and Carrero-Mantilla [71] used a processing degradation index (PDI) and used MFI values to quantify the extent of the degradation during reprocessing of PLA. The PDI was defined as follows:

(5) $$PDI=abs\left[\frac{\left({FI}_{0}+{FI}_{1}+...+{FI}_{n}\right)-\left(n+1\right){FI}_{0}}{(n+1){FI}_{0}}\right]$$

where *abs* means the absolute value, *n* the number of reprocessing cycles, and *FI_i_* the flow index in the reprocessing *i* (with *i* = 0 for raw polymer). MFI can thus be used to monitor and quantify degradation, although it only gives the flow characteristics at a single temperature and a single shear rate, which is often lower than the actual shear rates during polymer processing (e.g., injection molding). A viscosity curve contains more information about the rheological properties, but requires more training and knowledge to perform than an MFI measurement.

**Figure 8:**
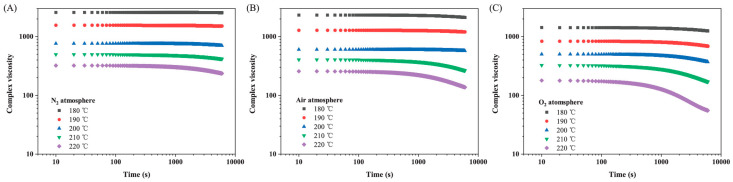
Time evolution of complex viscosity (η*) for degraded samples under different degradation conditions, (**A**) nitrogen atmosphere, (**B**) air atmosphere, and (**C**) oxygen atmosphere. Reprinted with permission from Ref. [32]. 2023, John Wiley and Sons.

All previously described viscosity- or MFI-measurements use a sample that is collected after processing on an injection molding machine or a single- or twin-screw extruder, which means that the sample is already degraded. A parallel plate rheometer is also reported in the literature to degrade the sample under specific processing conditions by performing an oscillatory time sweep analysis. During this measurement, neat PLA granules are used to study how materials change over time. Lin et al. [32] and Cuadri and Martín-Alfonso [33] exposed neat PLA to three different constant temperatures in either an air or nitrogen environment and studied how the rheological properties, such as η*, G′, and G″, evolved over time (see Figure 8). Nofar et al. [72] used the time to reach a 10% viscosity drop to compare how fast PLA and PBAT degraded. Other researchers [20,37,56,73] used the same test method to successfully study degradation through time, making oscillatory time sweep measurements an interesting method to study thermal, oxidative, and hydrolytic degradation. A disadvantage is that the mechanical stresses on the polymer chains are small in comparison to actual melt processing techniques such as extrusion and injection molding; therefore, studying thermomechanical degradation on a parallel plate rheometer is not ideal.

#### 3.1.3. Thermal Properties

**Figure 9:**
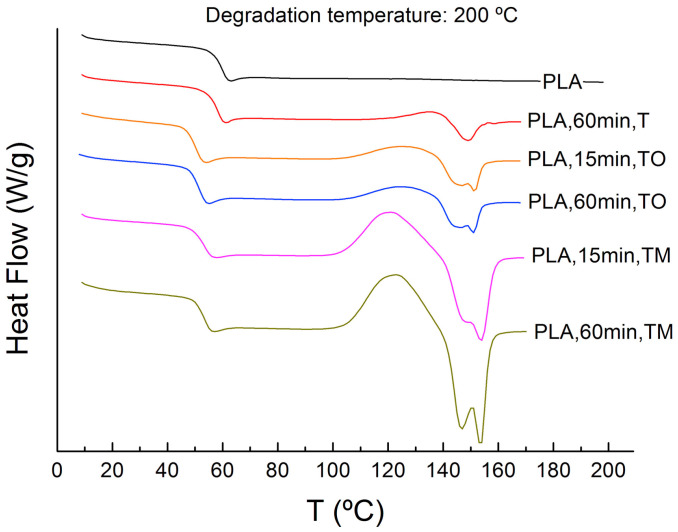
DSC curves corresponding to the second heating cycle for virgin PLA and selected degraded samples subjected to different degradation conditions (“T”, “TO”, and “TM” correspond to thermal, thermo-oxidative, and thermomechanical degradation, respectively). Reprinted with permission from Ref. [33]. 2023, Elsevier.

Differential scanning calorimetry (DSC) is a fast and cost-effective way to measure the thermal properties that are relevant to polymer melt processing, such as thermal transitions ($T_{g}$, $T_{m}$, crystallization temperature ($T_{c}$), and cold crystallization temperature ($T_{cc}$)), the crystallization behavior, and thermal heat capacity (see Figure 9). Most thermal properties depend on molecular weight, therefore DSC can be used to monitor process-induced degradation by analyzing the change in thermal properties after processing PLA.

Changes in thermal transitions caused by degradation are reported thoroughly in literature. Most researchers [26,50,52,54,59,61,62,69,70,71] did not conclude a significant change in $T_{g}$ before or after one or more melt processing cycles. However, a decrease in $T_{g}$ from 66.2 °C to 56.5 °C was reported in the work of Pillin et al. [53] after 7 reprocessing cycles. Nascimento et al. [68], Cuadri and Martín-Alfonso [33], and Badia et al. [74] also observed a decrease in $T_{g}$ (see Figure 9). The Flory-Fox equation links the $T_{g}$ to the molecular weight:

(6) $$T_{g}=T_{g}^{\infty }-\frac{K}{M_{n}}$$

in which $T_{g}^{\infty }$ is the $T_{g}$ at infinite molecular weight and K is a constant representing the excess free volume of the end groups for polymer chains. In addition to the dependence on the molecular weight, the $T_{g}$ is also influenced by the optical purity (ratio of L- to D-isomer) [74,75]. The different conclusions in the literature can be explained by different processing conditions on different PLA-grades with different optical purities. Thereby the extent of the degradation and thus the decrease in molecular weight will determine whether no significant change or a decrease in $T_{g}$ is observed. Since the change in $T_{g}$ from only one processing cycle is small, it can be concluded that the $T_{g}$ is not the best indicator to monitor process-induced degradation.

A second thermal property is the melting temperature, which is reported in most articles [17,26,52,53,59,65,68,70,76] to not significantly change after melt processing. Since it is known that the $T_{m}$ is also dependent on the molecular weight and the optical purity [75,77], it can be concluded that a larger decrease in molecular weight is required to result in a significant decrease in $T_{m}$. For example, in the work of Jain et al. [54], a decrease in $T_{m}$ was reported, from 190 °C to 178 °C, after processing it for 135 min in a cartridge 3D printer, linked to a reduction in $\overset{-}{M_{n}}$ from 145.0 kDa to 26.0 kDa. Also, in the work of Ragaert et al. [57], decreases in $T_{m}$ from 196 °C to 152 °C, and in $\overset{-}{M_{n}}$ from 139.9 kDa to 3.6 kDa, were reported after exposing PLLA to an isothermal heat treatment for 6 h. These long residence times are not realistic during melt processing techniques such as extrusion and injection molding. In addition, a double melting peak was reported in the works of Botta et al. [61], Badia et al. [74], Scaffaro et al. [62], and Cuadri and Martín-Alfonso [33], with the dominant peak at the higher temperature (see Figure 9). Since this double melting peak is not always present, realistic residence times during processing are limited to a few minutes and a melting peak is not always present due to a slow crystallization rate, $T_{m}$ is not an appropriate indicator to monitor degradation through processing.

The $T_{c}$ and $T_{cc}$ are important thermal properties for PLA to monitor the process-induced degradation, since all relevant studies [17,26,33,40,50,61,62,65,68,69,70,74,76] reported a decrease in $T_{cc}$ after melt processing (see Figure 9). In the work of Benvenuta-Tapia and Vivaldo-Lima [17], a reduction in $T_{cc}$ from 112 °C to 109 °C was observed after 1 processing cycle. Similar results were reported by Botta et al. [61], where the $T_{cc}$ progressively decreased from 110.9 °C to 106.2 °C after 5 extrusion cycles. This decrease in $T_{cc}$ was explained by chain scission taking place during melt processing, resulting in a decrease of the molecular weight, increasing the mobility of the polymer chains. In addition to a change in $T_{cc}$, two papers also reported a change in $T_{c}$. Lv et al. [52] reported a decrease in $T_{cc}$ from 103.0 °C to 97.1 °C and an increase in $T_{c}$ from 96.8 °C to 100.2 °C after 4 reprocessing cycles. In the work of Pillin et al. [53], a decrease in $T_{cc}$ was reported from 131.1 °C to 89.8 °C throughout 7 reprocessing cycles, whereas a $T_{c}$ was only observed from the 2nd reprocessing cycle on. $T_{c}$ increased from 94.9 °C to 97.7 °C between the 2nd and 7th processing cycle. As explained before, the molecular weight decrease from degradation results in higher chain mobility and easier crystallization, causing the crystallization during cooling to start at higher temperatures. It also explains why Pillin et al. [53] observed only a $T_{c}$ from the second reprocessing cycle on, since the crystallization is now fast enough to already start during cooling due to a large decrease in molecular weight. Only a minority of researchers observed crystallization during cooling (and thus $T_{c}$), since PLA is characterized by slow crystallization kinetics. The cooling rate during DSC measurements is often faster than the crystallization rate of PLA, resulting in only a cold crystallization peak during heating and no crystallization peak during cooling. The crystallization of PLA is not only affected by the molecular weight, but also by the optical purity. A low D-isomer content enhances crystallization, explaining why some authors do observe crystallization during cooling and others only observe cold crystallization. Therefore, $T_{cc}$ is an optimal thermal property to monitor the process-induced degradation, since cold crystallization occurs more frequently. In the less common case that crystallization during cooling occurs, $T_{c}$ can also be used as an indicator for degradation.

Since crystallization of PLA is known to be slow and it is affected by the decrease in molecular weight due to degradation, the degree of crystallinity is an interesting property to study. In the studied literature, two equations are reported to calculate $X_{c}$:

(7) $$X_{c}=\frac{∆H_{m}}{∆H_{m}^{0}}\times 100\%$$

(8) $$X_{c}=\frac{∆H_{m}-∆H_{cc}}{∆H_{m}^{0}}\times 100\%$$

with $X_{c}$ the degree of crystallinity, $∆H_{m}$ the melting enthalpy, $∆H_{cc}$ the cold crystallization enthalpy, and $∆H_{m}^{0}$ the theoretical melting enthalpy of 100% crystalline PLA, equal to 93.1 J/g. Publications in which Equation (7) was used [17,20,26,53,70] all concluded an increase in $X_{c}$, which confirms that degradation causes faster crystallization, although it is not clear if the crystals were formed during crystallization or cold crystallization, because $∆H_{cc}$ was not used in the calculation. Since cold crystallization occurred in all studied articles, the majority of articles took $∆H_{cc}$ into account and thereby used Equation (8) to calculate $X_{c}$. In the work of Scaffaro et al. [62], a gradual increase in $X_{c}$ from 3.6% to 4.8% was reported after 5 reprocessing cycles, whereas Lv et al. [52] observed an increase in $X_{c}$ from 24.3% after 1 processing cycle to 36.7% after 4 reprocessing cycles. Also, other researchers [59,61] concluded an increase in $X_{c}$ due to degradation. This means that crystallization occurred during the cooling cycle and the crystallization went proportionally faster with a decreasing molecular weight. In the works of Cuadri and Martín-Alfonso [33], Brüster et al. [50], Nascimento et al. [68], and Béltran et al. [76], it was concluded that $X_{c}$ was 0, since $∆H_{cc}$ and $∆H_{m}$ were found to be equal. This means that there was no crystallization during cooling, but only cold crystallization during the second heating cycle. An important remark is that when $X_{c}$ is equal to 0, it does not mean that the crystallization kinetics did not change due to degradation. To avoid misinterpretations, it is more interesting to only investigate $∆H_{cc}$ and $∆H_{c}$ instead of $X_{c}$ to monitor what the effect of degradation is on the crystallization kinetics, since most authors [26,50,69,74] reported an increase in $∆H_{cc}$ and no $∆H_{c}$ for degraded PLA. Other authors [52,53,59] reported an increase in $∆H_{c}$ and a decrease in $∆H_{cc}$, indicating more crystallization during cooling and less cold crystallization during heating. Both trends confirm that degradation results in faster crystallization kinetics, since the chain scission processes enhance chain mobility and the shorter chains can serve as nucleating agents. Since $X_{c}$ can be calculated and interpreted in different ways, it is not a good property to monitor the degradation of PLA during melt processing, as was confirmed by the work of Cuadri and Martín-Alfonso [33] (see Figure 9). Therefore, it is advised to study $∆H_{cc}$ to understand the extent of the degradation.

All aforementioned thermal properties with DSC were measured in the temperature range that is relevant to melt processing of PLA. With thermogravimetric analysis (TGA), it is possible to look at the thermal stability at higher temperatures. Benvenuta-Tapia and Vivaldo-Lima [17] found a decrease in the onset degradation temperature T_onset_ from 335 °C for unprocessed PLA to 332 °C (10% weight loss) after processing. Similar trends were found in the work of Zhao et al. [59], Nekhamanurak et al. [64], Cuadri and Martín-Alfonso [33], and Agüero et al. [26]. Thus, it can be concluded that processed PLA shows lower thermal stability, since chain scission creates shorter polymer chains that will decompose at lower temperature. Degradation does influence the thermal stability and can be used as an indicator, but it is less relevant for the temperature range of polymer processing.

#### 3.1.4. Mechanical Properties

The mechanical properties of a polymer product are crucial during its use phase, which emphasizes the importance of understanding the effect of degradation on the mechanical properties such as the elastic modulus (E), tensile strength (σ_t_), tensile strain at break (ε_b_), tensile stress at break (σ_b_), impact strength (IS), and hardness.

**Figure 10:**
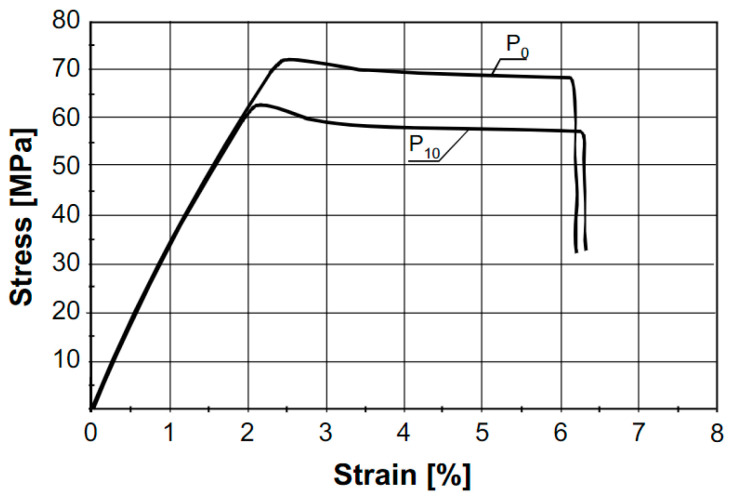
Stress-strain curves of PLA that was extruded one to ten times, with P_0_ a sample produced from original PLA and P_10_ a sample from PLA that was extruded ten times. Reprinted with permission from Ref. [69]. 2023, Elsevier.

The change in elastic modulus after melt processing was reported by multiple authors. Agüero et al. [26], Beltrán et al. [31], Zhao et al. [59], and Zenkiewicz et al. [69] observed no significant change in the stiffness of the processed PLA as illustrated in Figure 10. In the work of Pillin et al. [53], a decrease from 4493 MPa to 4223 MPa after 7 injection molding cycles was reported, which was confirmed by other researchers [50,52] describing a similar decrease in the stiffness. Badia et al. [74] reported no change in E in the 1st and 2nd reprocessing cycle, but a 28% decrease in E was observed between the 2nd and 5th reprocessing cycle. Scaffaro et al. [62] reported an increase in the stiffness up to the third reprocessing cycle, but a decrease between the third and fifth reprocessing cycle. The variety in conclusions was explained through the competition between two phenomena: on the one hand, a decrease in molecular weight and thus in entanglements resulting in a lower stiffness; on the other hand, an increase in $X_{c}$ leading to a higher stiffness. The balance between optical purity, Mw, and $X_{c}$ is crucial, which was confirmed in the work of Cosate De Andrade [55], who reported an increase in E from 2611 MPa of virgin PLA ($X_{c}$ 0%) to 3499 MPa after 2 extrusion cycles ($X_{c}$ 22%). Since all the research described above melt-processed different PLA-grades with different optical purities, there is no clear trend in how the stiffness will be affected.

**Figure 11:**
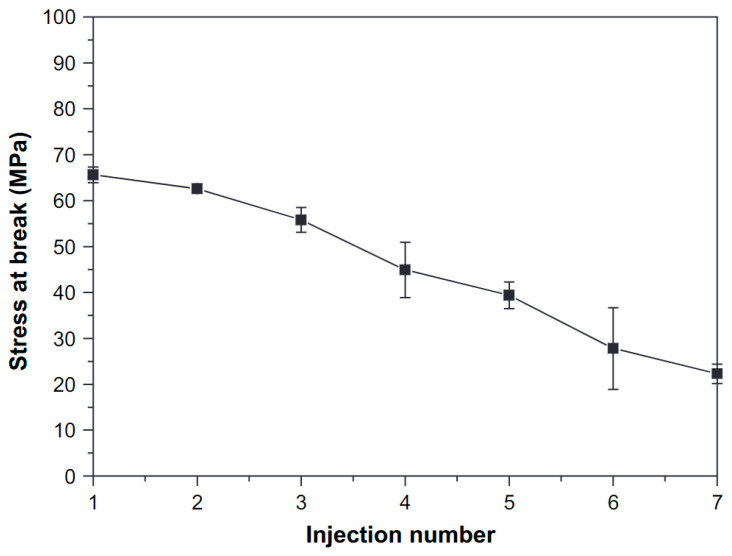
Stress at break (σ_b_) as a function number of injection molding cycles. Reprinted with permission from Ref. [53]. 2023, Elsevier.

In addition, σ_t_ and σ_b_ are often studied in literature. Zhao et al. [59] and Scaffaro et al. [62] reported that σ_t_ was not changed after one and five processing cycles, respectively. In the work of Cosate De Andrade et al. [55], a decrease in σ_t_ was reported from 63 MPa to 55 MPa (13%) after 2 extrusion cycles, which is a similar trend as that reported by Zenkiewicz et al. [69], where a decrease of 1.44 MPa (2%) in σ_t_ was observed after 1 extrusion cycle. About the change in σ_b_, all authors [26,50,53,69,74] came to the same conclusion: melt processing of PLA results in a decrease in σ_b_. Specifically, in the work of Pillin et al. [53], a decrease was observed from 66 MPa to 25 MPa after 7 injection molding cycles as illustrated in Figure 11. Both σ_t_ and σ_b_ are dependent on the molecular weight and thus the extent of the degradation: a decrease in the molecular weight means a lower number of entanglements and thereby a decrease of σ_t_ and σ_b_.

**Figure 12:**
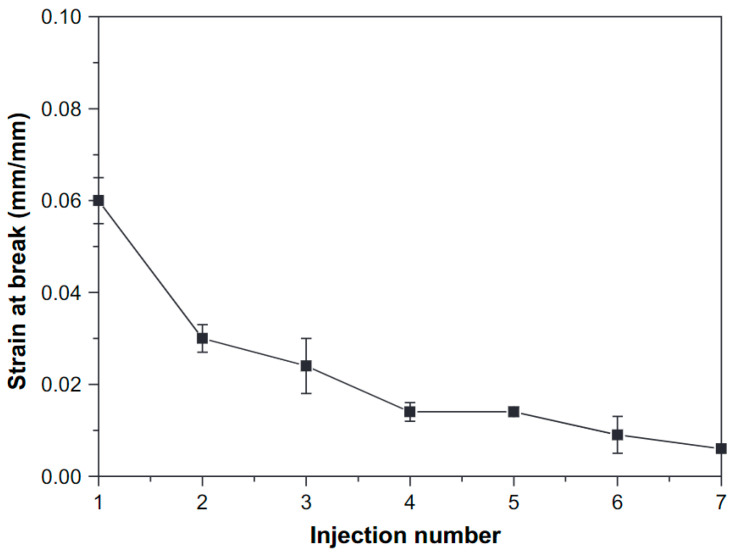
Strain at break (ε_b_) as a function number of injection molding cycles. Reprinted with permission from Ref. [53]. 2023, Elsevier.

In addition to stress and stiffness, ε_b_ was reported to decrease as a result of degradation, for example in the work of Lv et al. [52], where a decrease from 9% to 5% was observed after 4 reprocessing cycles, and Aguëro et al. [26], where ε_b_ decreased from 9.73% to 6.28% after 6 reprocessing cycles. The decreasing trend in ε_b_ was confirmed by other authors [53,55,59] as illustrated in Figure 12, although no change ε_b_ was reported in the works of Scaffaro et al. [62], Zenkiewicz et al. [69], and Badia et al. [74]. The combined effect of a decrease in Mw (less entanglements) and an increase in $X_{c}$ (easier crack propagation) due to degradation determines whether a decrease in ε_b_ is observed or not.

**Figure 13:**
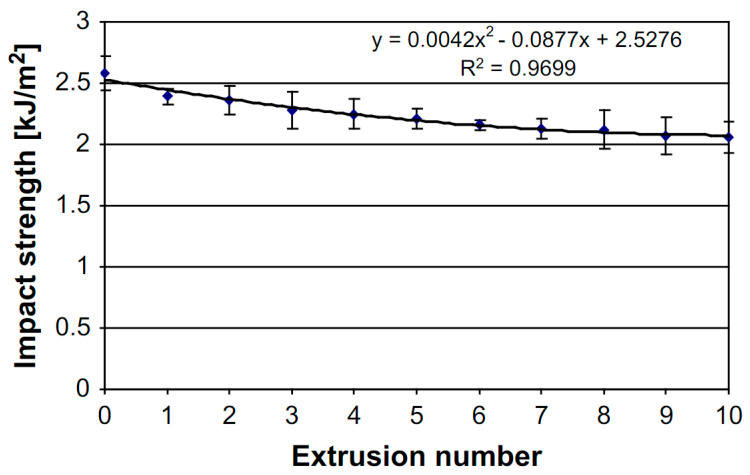
Impact strength (IS) as a function of the extrusion number. Reprinted with permission from Ref. [69]. 2023, Elsevier.

The IS of PLA is observed to decrease due to process-induced degradation. In the papers that investigated IS, all researchers reported a decrease due to chain scission. In the work of Zenkiewicz et al. [69], a decrease of 0.54 kJ/m^2^ was reported throughout 10 reprocessing cycles as illustrated in Figure 13, which is a decrease of 20.2%. The largest decrease of 0.19 kJ/m^2^ occurred in the 1st processing cycle and corresponded to a decrease of 7.4%. In the work of Agüero et al. [26], a decrease of 57.8 kJ/m^2^ to 31.1 kJ/m^2^ was observed, and a similar decreasing trend was reported by other researchers [50,55,74]. The decrease in Mw due to processing, linked to an increased chain mobility and an increase in $X_{c}$, creates a more brittle material. As a result of this, the energy absorption capacity is reduced, resulting in a lower IS.

Pillin et al. [53] used nanoindentation to investigate the effect of degradation on the hardness of PLA. A slight decrease from 295 MPa to 250 MPa was reported after 7 injection molding cycles and was also attributed to the chain scission process, confirmed by Perego et al. [78]. Also, in the work by Beltrán et al. [31] a decreasing trend in the indentation hardness was reported from around 266 MPa to 248 MPa (7%) due to reprocessing. Contradictory to these results, no significant change in the Shore D hardness was observed by Agüero et al. [26] after six reprocessing cycles.

Overall, it can be concluded that mechanical properties are less optimal to monitor process-induced degradation, since the extent of degradation determines the balance between the decrease in Mw, the increase in $X_{c}$, and the optical purity of the PLA used in the experiments. The complex balance between these competitive events determines whether a change in mechanical properties occurs. Compared to molecular weight measurements, rheological properties, and thermal properties, the mechanical properties are less straightforward to indicate process-induced degradation.

#### 3.1.5. Visual Properties

In the work of Agüero et al. [26] the effect of reprocessing on the color of PLA was determined, by measuring the L*, a*, and b* coordinates. A decrease in the L* (black–white) value, from 73.23 to 58.65, was reported between virgin PLA and PLA after 6 processing cycles. An increase in b* (blue–yellow) from 10.75 to 20.59 indicated yellowing of the PLA due to reprocessing. Yellowing was also reported in the works of Mysiukiewicz et al. [79] and Hopmann et al. [30]. The change in color can be observed visually, but can also be measured easily and can serve as an indicator for process-induced degradation. However, other properties provide more in-depth information about the polymer chains and the cause of degradation. The change in color due to degradation should be avoided at all times, since color is crucial during the production of commercial products.

#### 3.1.6. Chemical Structure

**Figure 14:**
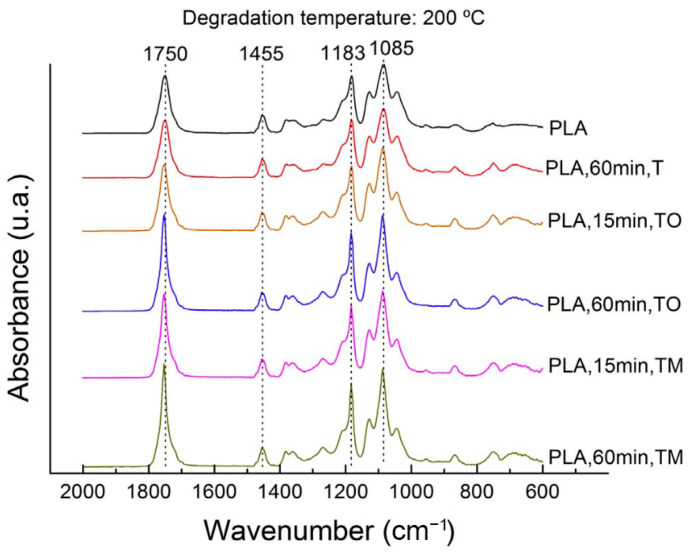
Attenuated Total Reflectance-Fourier Transform Infrared (ATR-FTIR) spectra for virgin PLA and selected degraded samples subjected to different degradation conditions (“T”, “TO”, and “TM” correspond to thermal, thermo-oxidative, and thermomechanical degradation, respectively). Reprinted with permission from Ref. [33]. 2023, Elsevier.

Process-induced degradation generates a change in the chemical structure, which can be measured with Fourier transform infrared spectroscopy (FTIR) as in the work of Cuadri and Martín-Alfonso [33], as illustrated in Figure 14. Since random chain scission is the predominant degradation reaction during photo-oxidation, thermo-oxidative degradation, and thermomechanical degradation, it was associated with the formation of anhydrides, carbonyl groups and/or carboxyl groups. These changes caused by degradation were mainly identified in the FTIR-spectra at 1085 cm^−1^ and 1183 cm^−1^ (asymmetric vibration of the ester group), and at 1750 cm^−1^ (carbonyl stretching). For all samples, also changes between 750 cm^−1^ and 650 cm^−1^ were observed, which was assigned to changes in crystallinity [80]. Analysis of the absorbance ratios of the aforementioned peaks allowed Cuadri and Martín-Alfonso [33] to conclude that nitrogen is required to prevent degradation since no significant changes in the peaks were visible for PLA subjected to thermal degradation. For PLA exposed to thermo-oxidative and thermomechanical degradation conditions, a significant increase in the absorbance ratio was concluded especially for the new carbonyl compounds at 1750 cm^−1^. In the work of Kister et al. [80], additional changes in crystalline regions were indicated at 921 cm^−1^ and 1293 cm^−1^. Studying the changes in chemical structure due to degradation was also done by Badia et al. [74]. Hence, FTIR is an interesting analysis to investigate process-induced degradation and to gain a better understanding of the degradation reactions and degradation products.

### 3.2. In-Line Measurements

The methods discussed above are all off-line measurements and can be used to determine the effect of processing on the final properties of the product, but they can be time-consuming and only give a snapshot view before and after processing. In-line measurements can also be used to monitor degradation in real time during processing, giving direct feedback. Ultra-violet/visible light (UV-vis) spectroscopy was used in the works of Wang et al. [47], Kesters et al. [81], and Malinauska et al. [82], since it is sensitive to color changes. It was concluded to be a suitable in-situ indicator for process-induced degradation of PLA and other polymers. Other techniques, such as near infrared (NIR), Raman, and ultrasound spectroscopy have also been used in the literature [32,80,83,84,85] and were able to successfully monitor the process-induced degradation in a continuous, non-invasive way.

In the work of Tuna and Ozkoc [18], the barrel of a micro-compounder was placed on a lever and was counter-balanced at the other end by a load cell, which was able to measure the vertical force during the extrusion process. The vertical force represented the melt viscosity of a polymer at a constant temperature and screw rotation speed, therefore enabling a comparison of the vertical force of virgin and processed PLA. Since the vertical force does not allow for calculation of the melt viscosity value due to the complex geometry of an extruder, it can only be used to compare materials during processing in a relative manner. Thus, it can be used as an indicator for process-induced degradation throughout the extrusion process, but it does not give additional information about the molecular weight or viscosity.

**Figure 15:**
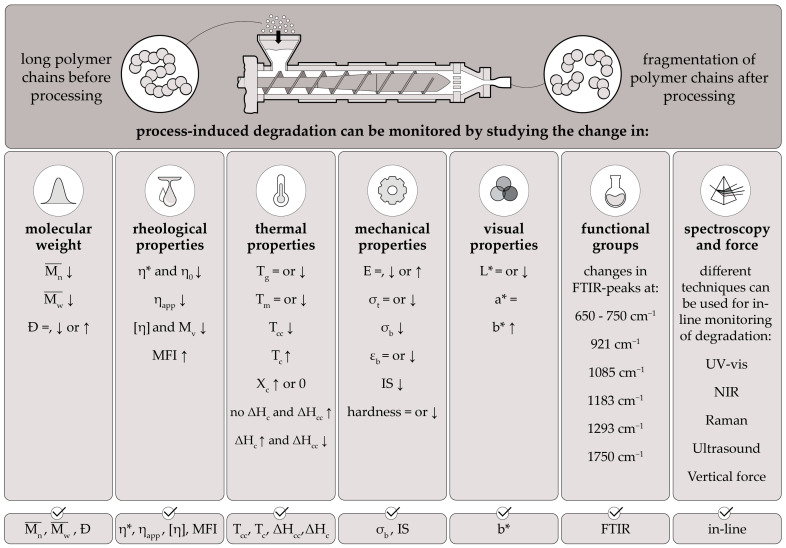
Overview on how degradation affects different properties and a selection of the properties (presented at the bottom row) that are eligible to monitor the process-induced degradation. $\overset{-}{M_{n}}$ ↓ [5,12,17,20,28,38,43,47,48,51,52,53,54,55,56,57,58,59]. $\overset{-}{M_{w}}$ ↓ [12,17,20,28,29,30,38,43,48,49,50,51,52,53,55,56,58,59]. Đ = [5,30,48,50,51,53], Đ ↓ [12,17,28,54,55] or Đ ↑ [49,52]. η* and η_0_ ↓ [17,20,30,33,50,59,60,61,62,63,64,65]. η_app_ ↓ [64,66]. [η] and M_v_ ↓ [18,20,38,58,61,65,67]. MFI ↑ [12,26,40,55,68,69,70]. T_g_ = [26,50,52,54,59,61,62,69,70,71] and T_g_ ↓ [33,53,68,74]. T_m_ = [17,26,52,53,59,65,68,70,77] or T_m_ ↓ [54,57]. T_cc_ ↓ [17,26,33,40,50,61,62,65,68,69,70,74,76]. T_c_ ↑ [52,53]. X_c_ ↑ [17,20,26,52,53,59,61,62,70] or X_c_ 0 [33,50,68,76]. No $∆H_{c}$ and $∆H_{cc}$ ↑ [26,50,69,74] or $∆H_{c}$ ↑ and $∆H_{cc}$ ↓ [52,53,59]. E = [26,31,59,69], E ↓ [50,52,53] or E ↑ [55,62]. σ_t_ = [59,62] or σ_t_ ↓ [55,69]. σ_b_ ↓ [26,50,53,69,74]. ε_b_ = [62,69,74] or ε_b_ ↓ [26,52,53,55,59]. IS ↓ [26,50,55,69,74]. Hardness = [26] or hardness ↓ [31,53,78]. L* = [79] or ↓ [26]. a* = [26,79]. b* ↑ [26,30,79]. Change in peaks at 650-750 cm^−1^ [33], 921 cm^−1^ [80], 1085 cm^−1^ [33], 1183 cm^−1^ [33], 1293 cm^−1^ [80] or 1750 cm^−1^ [33]. UV-vis [47,81,82]. NIR [80,84]. Raman [32,80,84]. Ultrasound [83,85]. Vertical force [18].

All possible methods to monitor PLA degradation discussed with the references are combined in Figure 15 in a concise manner.

## 4. Influence of Processing Variables on Degradation

The previous part of this review article discussed the different properties of PLA that can be used to monitor process-induced degradation. The following part will discuss how the processing parameters influence the extent of the degradation. A proper selection of the processing parameters can minimize process-induced degradation and therefore minimize the changes in properties of PLA, which is critical during industrial processing of PLA consumer products. An overview of the different papers that investigate the effects of different processing variables can be found in Table 1. All included articles comply with the criteria that processing occurs with industrial polymer processing techniques (e.g., single-screw extrusion, co-rotating twin-screw extrusion (TSE), injection molding), that the degradation during processing was studied (e.g., no biodegradation), and that neat PLA was processed (e.g., no fillers or additives).

### 4.1. Influence of Processing Temperature

**Figure 16:**
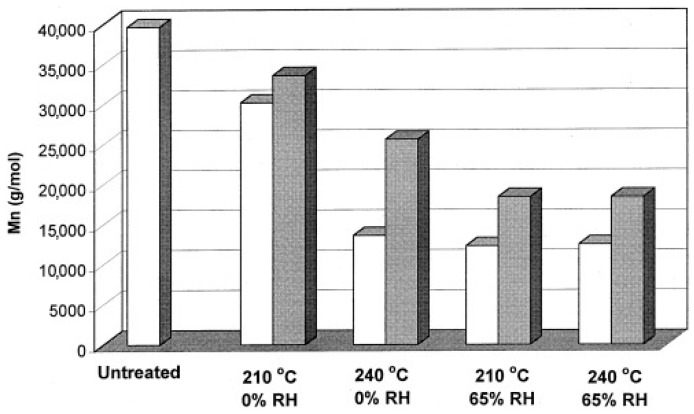
Averaged number-molecular weight ($\overset{-}{M_{n}}$) as function of processing conditions. White: 20 rpm; grey: 120 rpm. Reprinted with permission from Ref. [89]. 2023, John Wiley and Sons.

The influence of the processing temperature on the degradation is studied thoroughly in literature [5,19,27,39,47,79,86,87,88,89], with a uniform conclusion that an increase in temperature increases degradation. Gonçalves et al. [5] reported a decrease in $\overset{-}{M_{n}}$ from 138.0 kDa for unprocessed PLA to 132 kDa for PLA processed between 190 °C and 200 °C, whereas a decrease in $\overset{-}{M_{n}}$ to 125.0 kDa was found for PLA processed between 200 °C and 210 °C. In the work of Mysiukiewicz et al. [79], 4 different PLA-grades were processed on a twin-screw extruder at temperatures between 180 °C and 260 °C. It was observed that no significant degradation reactions occurred at temperatures up to 200 °C for the different PLA-grades, but that the molecular weight decreased significantly at higher temperatures. During processing PLA 4032D at 180 °C, η_0_-values of 2088 Pa·s and 2166 Pa·s were reported at 50 rpm and 250 rpm respectively, whereas the difference was much larger when processed at 260 °C, where 183 Pa·s and 397 Pa·s were reported at 50 rpm and 250 rpm, respectively. The results of this study emphasize that different processing variables may have an interaction effect. The importance of possible interactions between the processing temperature and other processing variables was confirmed in the work of Taubner and Shishoo [89], as seen in Figure 16. For PLA processed at 210 °C, the $\overset{-}{M_{n}}$ decreased from 33.6 kDa to 30.9 kDa when reducing the screw rotation speed from 120 to 20 rpm. During processing PLA at 240 °C, $\overset{-}{M_{n}}$ was measured to be 25.6 kDa and 13.6 kDa when processed at 120 and 20 rpm, respectively. Overall, it can be concluded that working at high processing temperatures should be avoided, since thermal degradation is causing chain scission. Additionally, the processing temperature has potential cooperative effects with several processing variables, which should be taken into account when these are evaluated.

### 4.2. Influence of Screw Rotation Speed

**Figure 17:**
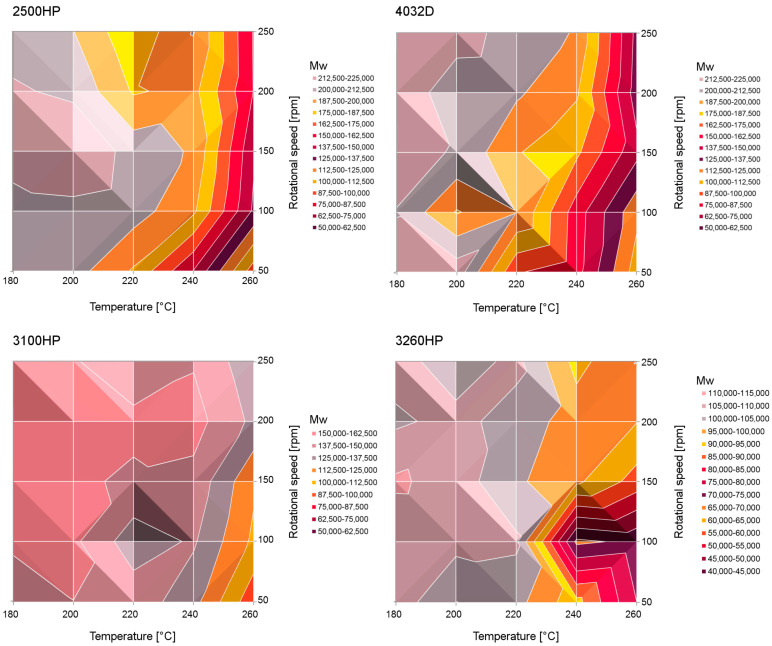
Molecular weights as a function of processing temperature and rotational speed for four PLA 2500HP, PLA 4032D, PLA 3100HP, and PLA 3260HP [79].

Contradictory results about the influence of screw rotation speed on the degradation are found in the literature. Most articles [79,86,89,90] concluded that an increase in screw rotation speed resulted in a smaller decrease in molecular weight (see Figure 16 and Figure 17), which can be explained by a shorter residence time. At a higher screw rotation speed, the residence time and thus the exposure of PLA to high temperature and shear inside the extruder is shorter, resulting in reduced degradation. In the work of Atalay et al. [86], a lower complex viscosity was reported when amorphous PLA was processed at 50 rpm than at 100 rpm, both at a processing temperature of 150 °C. In addition, Aldhafeeri et al. [90] reported η_0_-values of 4806 Pa·s at 400 rpm and 5332 Pa·s at 1000 rpm due to the longer residence time at lower screw rotation speeds. As indicated before in the works of Mysiukiewicz et al. [79] and Taubner and Shishoo [89], it is important to take the processing temperature into account when evaluating the effect of screw rotation speed. Other researchers concluded the opposite result, namely a decrease in the screw rotation speed resulting in less degradation. For example, in the work of Wang et al. [47], it was concluded that an increase in the screw rotation speed resulted in more severe degradation. On the one hand, a higher screw rotation speed causes more shear and elongational deformation, which leads to an additional heating of the melt. On the other hand, an increasing screw rotation speed results in a shorter residence time. The authors conclude that the additional mechanical impact had a larger effect than the reduction in residence time, thereby resulting in more severe degradation when the screw rotation speed increased. Similar results were observed in the work of Kosmalska et al. [19].

### 4.3. Influence of Screw Configuration

In several research papers, the influence of the screw configuration on the degradation was reported. In the work of Kosmalska et al. [19], two screw configurations on a twin-screw extruder were used to process PLA. The difference between both was the number of kneading and mixing elements, with a less demanding screw configuration containing two elements and a more demanding screw configuration containing four elements. The screw configuration was found to be of fundamental importance, since more kneading and mixing elements complicated processing and increased both the residence time of the melt inside the extruder as well as the shear stresses on the PLA. It was observed that PLA processed with the more demanding screw configuration always resulted in a larger decrease in molecular weight (while processing at different temperatures and screw rotation speeds) when compared to using the less demanding screw configuration. More specifically, when PLA was processed at 210 °C at 600 rpm, the molecular weight decreased 16.5% with the less demanding screw configuration, and 28% with the more demanding screw configuration. In addition, in the work of Aldhafeeri et al. [90] it was observed that the use of kneading blocks in both twin-screw extrusion and quad-screw extrusion (QSE) increased the degradation. While processing PLA at 400 rpm in a twin-screw extruder, a η_0_ of 4806 Pa·s was reported when no kneading blocks were used whereas a η_0_ of 4356 Pa·s was reported when the screws contained kneading blocks.

### 4.4. Influence of Throughput

**Figure 18:**
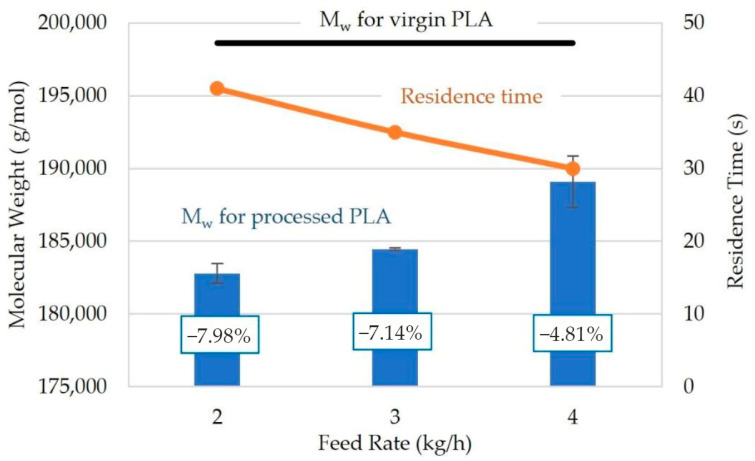
Weight-average molecular weight ($\overset{-}{M_{w}}$) and residence time for PLA processed using quad-screw extrusion (QSE) at a screw speed of 400 rpm and with screw configuration 2 (no kneading blocks). The boxed values represent reductions in molecular weight with respect to the molecular weight of the virgin PLA [90].

In the work of Aldhafeeri et al. [90], the influence of the throughput during QSE was investigated and it was observed that a higher throughput resulted in less degradation, as illustrated in Figure 18. A throughput of 4 kg/h caused a molecular weight decrease of 4.81%, where the decreases were 7.14% and 7.98% at a throughput of 3 kg/h and 2 kg/h, respectively. Less degradation is caused at higher throughput since the residence time is shorter. Similar research was conducted by Wang et al. [47], who varied the throughput during TSE of PLA. It was observed that decreasing the throughput caused an increase in degradation because the lengthened residence time allowed for a longer duration for degradation reactions to occur. At a screw rotation speed of 400 rpm, a throughput of 0.61 kg/h resulted in a residence time of 2.9 min, whereas a residence time of 8 min was obtained when a throughput of 0.2 kg/h was used. It is important to emphasize that the geometry of the equipment (screw diameter D and the length-to-diameter ratio L/D) will directly affect the outcome of the experiment.

### 4.5. Influence of Mixing Time

The influence of screw rotation speed (Section 4.2), screw configuration (Section 4.3), and throughput (Section 4.4) on the extent of the degradation was determined and explained by the residence time of the PLA melt inside the processing equipment. An internal mixer, usually consisting of two screws enclosed in a mixing chamber, allows one to precisely select and control the mixing time on a laboratory scale. In the work of Le Marec et al. [88], the effect of a mixing time of 10, 20, or 30 min at 40 rpm was studied on the molecular weight. It was concluded that a longer mixing time caused more chain scission since the melt is exposed longer to high temperatures, stresses, and moisture, resulting in a decrease of the molecular weight. Additionally, in the work of Signori et al. [39], an internal mixer was used to control the mixing time to exactly 10 min at a fixed screw rotation speed of 50 rpm, which is not possible with other processing equipment since the geometry and screw geometry will determine the residence time of the melt inside. Taubner and Shishoo [89] used a variant of an internal mixer, which is a miniaturized extruder containing two screws. Therefore, the residence time is (similar to industrial processing techniques) defined by the selected screw rotation speed.

### 4.6. Influence of Moisture Content

The influence of the moisture content in PLA during processing is crucial, since residual moisture will cause hydrolytic degradation especially at high temperatures. In the work of Le Marec et al. [88], both dried (<200 ppm) and undried (around 1500 ppm) PLA was processed. Drying PLA granules before processing resulted in a decrease in the degradation rate constant by a factor of 2, highlighting the importance of avoiding moisture in PLA before processing. Signori et al. [39] confirmed this with experiments on an internal mixer, since it was observed that drying PLA prior to processing partially prevented degradation. In the works of Atalay et al. [86] and Taubner and Shishoo [89], the PLA granules were conditioned at 25%, 30%, and 60% RH at 60 °C for 3 days, or 65% RH at 20 °C for 24 h prior to processing, respectively. Atalay et al. [86] observed similar degradation for amorphous PLA (aPLA) containing 25% and 30% RH that was processed at 190 °C, but severe degradation occurred during processing of aPLA conditioned at 60% RH. Taubner and Shishoo [89] concluded that the presence of moisture in PLA affected the molecular weight decrease significantly at 210 °C, but that thermal degradation was dominant at 240 °C (see Figure 16), meaning that moisture content does not contribute further to the degradation processes. Overall, moisture during processing should be avoided at all times to minimize deterioration of the properties due to hydrolytic and thermal degradation.

### 4.7. Influence of PLA-Grade

In the work by Atalay et al. [86], a semi-crystalline and an amorphous PLA-grade, containing 12% and 0.5% D-isomer respectively, were processed during TSE. It was concluded that the percentage D-isomer had no influence on the thermal degradation since both showed similar viscoelastic behavior over time during processing. Paakinaho et al. [91] investigated the effects of three PLA-grades with a different initial molecular weight on the degradation during melt spinning. It was concluded that a higher initial molecular weight caused more degradation, which was explained by long polymer chains undergoing degradation in the extruder due to high shear stresses until the molecular weight and viscosity was low enough to withstand further degradation. The low molecular weight PLA (around $\overset{-}{M_{w}}$ 100.0 kDa) did not undergo chain degradation during extrusion, while the chain length of the middle ($\overset{-}{M_{w}}$ 270.0 kDa) and high molecular weight PLA (around $\overset{-}{M_{w}}$ 348.0 kDa) decreased 27% and 55%, respectively. In addition, in the work of Mysiukiewicz et al. [72], four different PLA-grades were used with a different MFI and thus a different molecular weight (see Figure 17). Similar conclusions about the initial molecular weight and MFI were found: PLA-grades with a low initial molecular weight (high MFI) were less prone to degradation, which was explained by the shorter residence time in the plastifying unit during TSE. Where processing PLA with an MFI of 65 g/10 min resulted in a 4-time decrease in η_0_, the η_0_ decreased 20 times when PLA with an MFI of 8 g/10 min was processed.

### 4.8. Influence of Processing Atmosphere

Signori et al. [39] investigated the effect of the processing atmosphere on the degradation of PLA by performing experiments under a nitrogen or air environment with a laboratory internal mixer. It was concluded that processing under a nitrogen atmosphere removed oxygen and thereby caused less degradation of the polymer. Minimizing degradation by working under a nitrogen atmosphere was even more effective than the effect of pre-drying PLA, indicating that thermo-oxidative degradation reactions are more important than hydrolytic degradation processes.

### 4.9. Influence of Processing Technique

**Figure 19:**
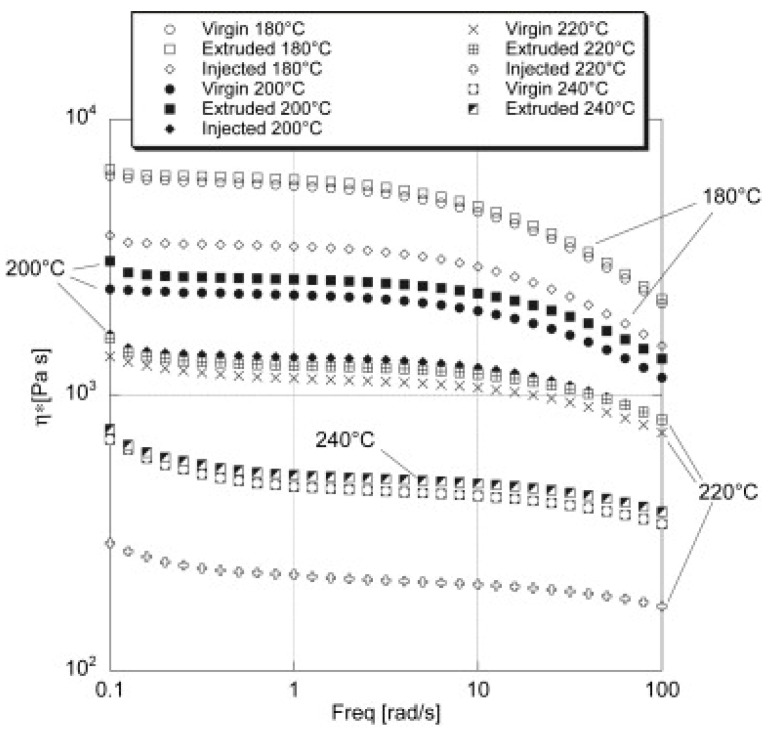
Complex viscosity (η*) of virgin and processed samples at different temperatures. Reprinted with permission from Ref. [92]. 2023, Elsevier.

Multiple researchers investigated how the selected melt processing technique affects degradation. In the works of Carrasco et al. [43], Cifuentes et al. [67], and Pantani et al. [92], the degradation due to SSE and IM was compared. Cifuentes et al. [67] concluded a decrease in molecular weight of 8% PLDA via IM, whereas a decrease of 6% was observed for PLLA after SSE. It should be noted that a clear comparison between IM and SSE was obstructed in this work by the use of two different PLA-grades and processing variables being selected differently. Pantani et al. [92] reported more degradation during IM than during SSE as illustrated in Figure 19, which may be explained by an average residence time of the melt at high temperature of 1 min during SSE and 15 min during IM. In the work of Carrasco et al. [43], PLA processed via IM was compared with PLA that was both extruded and injection molded SSE + IM. The $\overset{-}{M_{n}}$ of unprocessed PLA (69.3 kDa) decreased to 48.2 kDa during IM, and a further decrease was observed during IM and SSE to a $\overset{-}{M_{n}}$ of 42.9 kDa. A similar study was performed by Scoponi et al. [93], where unprocessed PLA was compared with PLA that was processed via IM, TSE, and both co-rotating twin-screw extrusion and compression molding (TSE + CM). It was observed that the $\overset{-}{M_{n}}$ of unprocessed PLA (74.1 kDa) decreased to 52.8 kDa after IM, 39.2 kDa after TSE, and 22.5 kDa after TSE + CM. In this research, TSE of PLA caused more degradation than IM due to a longer residence time and higher shear stresses in the TSE process. Scaffaro et al. [94] compared processing virgin PLA ($\overset{-}{M_{n}}$ 113.3 kDa) by using SSE, TSE, and a counter-rotating twin-screw extruder (TSC), causing a decrease in $\overset{-}{M_{n}}$ to 69.1 kDa, 81.0 kDa, and 59.4 kDa, respectively. TSE caused the least degradation, whereas the TSC resulted in more severe degradation. Two different causes were identified to be responsible: different residence times (60 s for TSE, 90 s for SSE, and 100 s for TSC) and different shear stresses during the extrusion processes. In the work of Aldhafeeri et al. [90], the effects of TSE and QSE on the degradation were investigated and it was observed that QSE resulted in more degradation. For example, η_0_ after TSE (no kneading blocks, 400 rpm) was 4806 Pa·s, whereas after QSE, η_0_ was 4276 Pa·s due to a longer residence time. Overall, it should be concluded that a comparison between processing techniques can only be made when the residence time, the thermal history, and the shear history of the melt is similar. Therefore, a proper selection of the processing variables during the experiments is crucial. This emphasizes also that the extent of the degradation is dependent on the used equipment and highlights why it is important to express the degradation in terms of the four fundamental parameters (residence time, processing temperature, shear rate, and moisture content) instead of equipment specific processing variables that cannot be compared.

## 5. Additives for Melt Stabilization of PLA

The previous sections discussed how processing variables affect the extent of the degradation, emphasizing the sensitivity of PLA to hydrolytic, thermal, oxidative, and thermo-mechanical degradation. In order to minimize deterioration of properties, multiple methods are discussed in the literature to enhance the stability of PLA: the use of chain extenders, antioxidants, and fillers.

**Figure 20:**
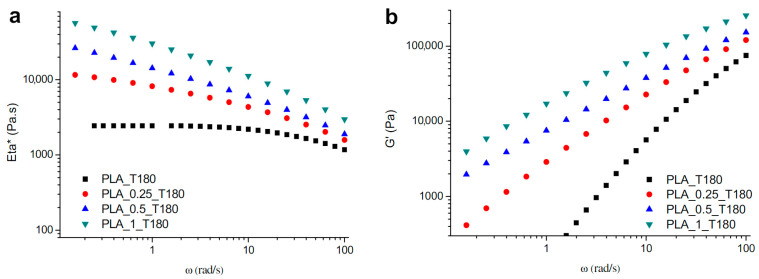
The complex viscosity (η*) (**a**) and the storage modulus (G′) (**b**) angular frequency dependence at 180 °C for neat and modified PLA with chain extender after reaching the equilibrium state. Reprinted with permission from Ref. [20]. 2023, Elsevier.

A first method that should be considered is the use of chain extenders, that contain functional groups that can react with the end groups of PLA chains. [40] Chain extenders are able to reconnect cleaved chains and thereby increase the chain length, enhance the melt strength, and increase the viscosity. Bifunctional chain extenders contain two functional groups and can react with two end groups, resulting in a linear structure. A branched or crosslinked structure can be obtained by using multifunctional chain extenders containing more than two functional groups. In the work of Al-Itry et al. [20], commercially available multifunctional epoxide (Joncryl ADR-4368, supplied by BASF) was incorporated in PLA. $\overset{-}{M_{w}}$ improved from 94 kDa for PLA without chain extender processed at 180 °C to 129 kDa (0.25 wt%), 159 kDa (0.50 wt%), and 185 kDa (1.00 wt%) for PLA with Joncryl. The increase in $\overset{-}{M_{w}}$ is linked to an increase [η], G′ and η* (see Figure 20), which confirmed the improved thermal stability due to the formation of branching chains. The same chain extender was also used in other studies [18,38,55,87,95] (see Figure 5), in which similar trends in molecular weight and viscosity were obtained. Additionally, other chain extenders and branching agents were investigated in the literature to enhance the stability of PLA during processing: polycarbodiimide (PCDI) [37,87,95], tris (nonylphenyl) phosphite (TNPP) [95], dicumyl peroxide (DCUP) [37,58], 1,4-phenylene diisocynate (PDI) [18], pyromellitic dianhydride (PMDA) [38], and hexamethylene diisocynate (HDI) [38]. Although the chain extension reactivity and efficiency are linked to the type of chain extender or branching agents used, all studies concluded an improvement of the molecular weight.

**Figure 21:**
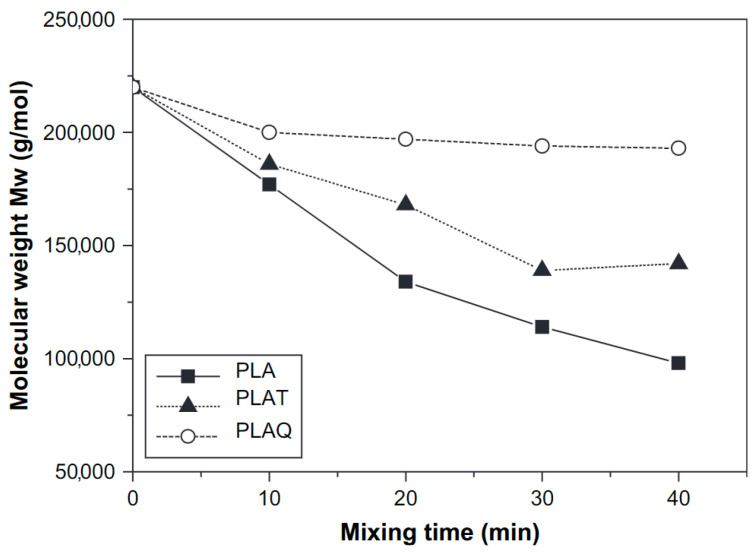
Molecular weights for PLA, PLAT (tropolone) and PLAQ (quinone) as a function of mixing time. Reprinted with permission from Ref. [53]. 2023, Elsevier.

A second method to minimize degradation during processing is by adding antioxidants. Antioxidants are indispensable additives that act by neutralizing the radicals. Pillin et al. [53] studied the effects of tropolone, p-benzoquinone, and hydroquinone as antioxidants on the degradation of PLA. Quinone strongly stabilized PLA and the molecular weight was concluded to stay quite stable with mixing time (decrease from 220 kDa to around 200 kDa after 40 min), as illustrated in Figure 21. Tropolone was found to be less active as antioxidant, since a decrease was reported from 220 kDa to around 100 kDa after 40 min mixing time. In addition, in the work of Amorin et al. [27], four different antioxidants were investigated (Irganox 1010, Irganox 1076, Irganox B 900, and Irgafos 168, supplied by BASF), concluding that the synergetic effect of primary and secondary antioxidants was a suitable way to stabilize the PLA, but the stabilizing effect was suppressed by the presence of nonradical reactions that occurred during the exposure of PLA to higher temperatures and shear.

**Figure 22:**
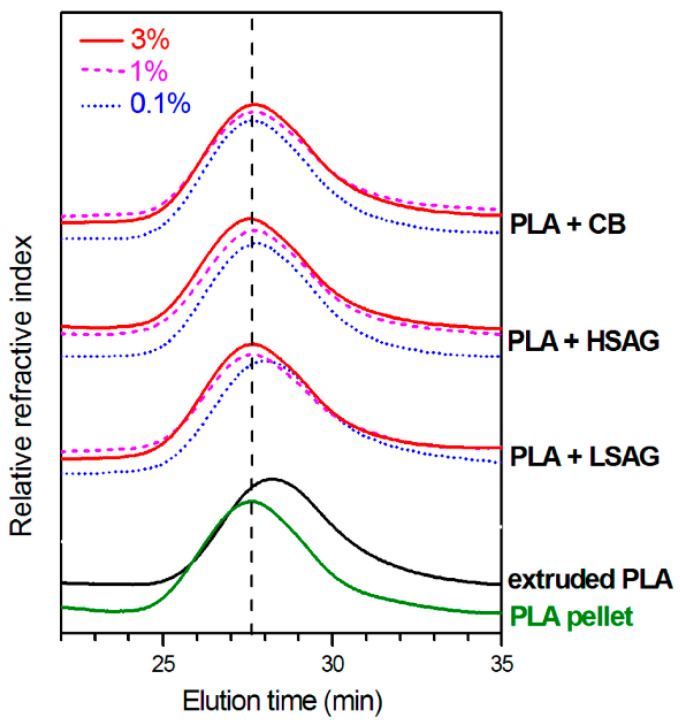
GPC curves of PLA pellets, extruded PLA and extruded PLA compounds with 0.1 wt%, 1 wt%, and 3 wt% of different carbon fillers LSAG, HSAG, and CB [56].

A third method to enhance the stability of PLA is by incorporating organic or inorganic (nano)fillers. Introducing fillers to PLA allows for improvement of the thermal stability and an increase in the typically slow crystallization rate [28,40,96]. In the work of Araújo et al. [97], the effects of three different types of montmorillonite (Mt) on thermal stability and crystallization were studied. GPC results of PLA and the 3 nanocomposites before and after 120 h of thermo-oxidative degradation showed an improvement of the thermal stability for 1 type of Mt (Dellite 43B, supplied by Lavriosa Mineraria). The different behaviors of the clay minerals were explained by their chemical compositions and structures. In the work of d’Urso et al. [56], different carbon fillers (low-surface-area graphite (LSAG), high-surface-area graphite (HSAG), and carbon black (CB)) were studied as possible stabilizers. Rheological time sweep measurements and GPC-analysis confirmed the melt stabilization of PLA after extrusion at 200 °C for all carbon fillers. A molecular weight reduction of about 25% for neat PLA was measured, while the molecular weight remained unaltered after compounding PLA with 0.1 wt% of HSAG or CB (see Figure 22). The time sweep measurements at 200 °C for 3 h, confirmed the ability of carbon nanofillers to stabilize PLA toward degradation reactions.

## 6. Discussion and Conclusions

The sensitivity of ester groups in PLA to high temperature, moisture, and shear stress forms a major issue in popular melt processing techniques such as injection molding and extrusion. The thermal, thermomechanical, and hydrolytic degradation result in an unwanted rapid loss of molecular weight and deterioration of the polymer properties. Although process-induced degradation cannot be fully avoided, it is crucial to understand how the selection of the processing variables affects the polymer chains to minimize degradation. Suppressing the degradation will benefit industrial processers of PLA, since they can reduce production cost, the amount of post-production waste, energy losses, and the use of raw materials, to deliver high quality and reproducible polymer products. Through the investigation of the current literature on process-induced degradation, it was possible to determine how degradation of PLA can be monitored and how the selection of different processing variables affects the extent of the degradation during melt processing.

It is important to understand which properties are altered as a result of melt processing and thus can serve as indicators to monitor the degradation of PLA. The molecular weight distribution and thereby also the rheological properties directly linked to the molecular weight, were concluded to be the best suited properties to measure and understand PLA degradation. A general trend was reported: processing PLA results in a decrease in $\overset{-}{M_{n}}$ and $\overset{-}{M_{w}}$ due to chain scission because of different degradation reactions. The shorter polymer chains are linked to a decrease in viscosity (η_0_, η*, η_app_ and [η]) and an increase in MFI. In addition, the discoloration and the change in functional groups studied with FTIR were concluded to be good indicators for degradation, although they are less often studied in literature on this topic. It is more complex to use thermal and mechanical properties to monitor the degradation, since the decrease in molecular weight enables easier crystallization and can thereby result in an increase in the degree of crystallinity. In addition, the degree of crystallinity is also influenced by the optical purity of the PLA that was used in the different studies. If and how strong melt processing will alter the thermal and mechanical properties is a complex balance between these three events affecting each other. This complexity emphasizes that it is crucial to be aware that the extent of the degradation, and thus the change in properties, is dependent on which PLA-grades and processing equipment were used and which processing variables were selected.

Therefore, the third section of this review paper described the influence of different processing conditions on the process-induced degradation of PLA. As expected, a similar trend was found by different authors studying the processing temperature and moisture content: increasing the moisture content and processing temperature results in more degradation and thus a larger decrease in molecular weight. Chain scission reactions linked to hydrolytic and thermal degradation explain the importance of processing at a temperature below 200 °C and drying PLA prior to melt processing. The conclusions about all other investigated processing conditions (screw rotation speed, screw configuration, the used melt processing technique, and the throughput) are often contradictory. Some authors concluded that increasing the screw rotation speed caused less degradation, whereas others reported more degradation. This stresses the importance of expressing the degradation in terms of the four independent fundamental parameters that directly govern the extent of degradation (moisture content in the polymer, processing temperature, residence time, and shear) instead of other processing variables. Multiple processing variables contribute to the total shear on the material, such as the screw rotation speed and the screw configuration that may contain kneading and mixing elements. On the one hand, increasing the screw rotation means that the shear stresses will be higher and additional heating will occur due to shearing of the material, resulting in more thermomechanical and thermal degradation. On the other hand, increasing the screw rotation speed reduces the residence time and thus reduces the time that the melt is exposed to high temperatures and shear, which will reduce the amount of degradation. Also, shear heating results in a decrease in viscosity and thus less shear stresses on the melt. How the different processing conditions will affect the degradation of PLA depends on the balance between the four fundamental parameters. Overall, it can be concluded that high shear stresses should be avoided and that the residence time should be as short as possible, but it is important to realize the difficulty of translating this to ideal processing conditions since these are dependent on the equipment used in the experiments of the current literature.

In the last part of this review paper, different methods were presented to enhance melt stabilization of PLA. Chain extenders and branching agents reconnect cleaved chains and thereby increase the molecular weight, resulting in an improvement of the thermal stability. Antioxidants act by neutralizing radicals and stabilize the degradation rate and molecular weight. Thermal stability is also obtained by adding (in)organic fillers to the melt, which also enhances the crystallization rate.

The degradation of PLA is investigated thoroughly, since the degradation reactions and kinetics are known in depth, and it is considered a significant challenge. Researchers and industrial processors are aware that PLA is sensitive to degradation, meaning that they have to pay attention during melt processing to select the processing conditions carefully. Although the general trends on how processing affects degradation are discussed in the current literature on this topic, multiple pathways are identified that may be of interest in future work:

- Comparing results from different studies is difficult, since results are equipment-dependent. The size of the extrusion screw (L/D ratio), the screw geometry (length and depth of different zones), and the screw configuration (use of kneading and mixing elements) have a large effect on the shear loading and residence time of the material inside the extruder. Also, additional shear heating and thus thermal loading on the material should be considered. In the future, it would be meaningful to express total degradation in terms of the four fundamental parameters instead of equipment specific processing variables. This means that the combination of processing variables should be translated towards a total sum of the shear stresses, the final residence time of the melt inside the equipment, the final temperature of the melt (which will be slightly higher than the selected processing temperature due to shear heating), and the moisture content of PLA during processing. By translating all processing variables to fundamental variables, it will be possible to compare the results of multiple studies that used different settings and equipment.
- Not all authors take into account interaction effects between processing variables. The work of Mysiukiewicz et al. [79] concluded that the screw rotation speed had no significant effect on the molecular weight decrease at temperatures below 200 °C, but it plays an important role at higher temperatures up to 260 °C. This emphasizes the importance of investigating the cumulative effects between processing variables instead of only looking at one processing variable at a time, which is often the case in current literature. In future work, it is important to take into account both the individual and interaction effects of the selected processing variables.
- Companies and researchers who are new to working with PLA, are often aware of the degradation occurring during melt processing, but do not know how to properly select the processing conditions that minimize the molecular weight decrease. The same difficulty arises when a new PLA-grades are selected and processed. The general trends on how the processing conditions affect degradation are understood in the literature, but the advice of different researchers on the correct set of processing conditions are not applicable since other processing equipment with a different geometry is used. In future work, it will be interesting to set up a quantitative degradation model that is able to predict how large the molecular weight decrease will be and that is generally applicable for different types of equipment. This requires the model to be based on the four fundamental parameters determining the degradation. If a polymer processing company wants to process PLA in the future, it can then input the desired set of processing conditions in the model, which then calculates the expected decrease in molecular weight. By varying the input parameters in the model, the effects of variables can then be estimated and the desired processing conditions can be tuned to deliver the lowest extent of degradation possible. It can help companies to overcome an expensive trial and error phase in production, since it can save time to find the optimal settings and can reduce the amount of industrial PLA waste.

This literature review paper gives an overview on the available studies that discuss different properties that can be used to monitor process-induced degradation of PLA and the effects of different processing variables on the extent of the degradation. The fundamental knowledge about PLAs sensitivity to degradation is understood well in the current literature and is widely recognized as an issue during polymer processing, although some interesting pathways have been defined for future work. The usage of PLA and other biobased polyesters is expected to increase over the coming years in a large variety of applications, due to more environmental awareness by both companies and customers. Further investigation of the effect of processing variables will be crucial to prevent deterioration of the PLA properties and to produce high quality products. Understanding and predicting the effects of processing variables on the degradation will become crucial to use the available resources in a responsible way and support the polymer industry in producing them in an effective way.

## Figures and Tables

**Table 1:** Overview of articles that investigate the effects of processing variables on the degradation (SSE = single-screw extrusion, TSE = co-rotating twin-screw extrusion, LIM = laboratory internal mixer, IM = injection molding, CM = compression molding, QSE = quad-screw extrusion, MSE = melt-spinning extruder, TSC = counter-rotating twin-screw extrusion).

Authors	Grade	Process	Variables	Conclusions
Gonçalves et al. [5]	PLA 2500HP ^1^	SSE	Temperature (190–200 °C, 210–220 °C)	- Decrease in initial molecular weight observed at both temperature ranges, being more severe at higher temperatures - Higher processing temperatures cause more thermal degradation and thus chain scission of the PLA chains during extrusion
Kosmalska et al. [19]	PLA LX 175 ^2^	TSE	Screw configuration (less, more demanding) Temperature (210, 230, 250 °C) Screw rotation speed (200, 400, 600 rpm)	- Screw with a greater number of kneading-mixing zones, high temperatures, and high screw rotation speed results in strong degradation and thereby a reduction in viscosity and molecular weight - Kneading-mixing zones elongate the residence time and cause more shear stresses, a high temperature causes more thermal degradation, and a high screw rotation speed causes more shear and elongational deformation - Screw configuration is defined as the most harmful element influencing a deterioration of the properties
Amorin et al. [27]	Commer. PLA ^3^	SSE	Temperature (210, 220, 230 °C)	- The increase in MFI and thus decrease in molecular weight due to degradation after five SSE cycles is the largest at the highest temperature, emphasizing the importance of thermal degradation
Signori et al. [39]	PLA 2002D ^1^	LIM	Temperature (150, 170, 190, 200 °C) Moisture content (dried, undried) Processing atmosphere (nitrogen, air)	- Increasing the temperature causes a reduction in viscosity of the PLA due to thermal degradation - All combinations of drying PLA, processing under nitrogen, or a combination of both, shown to prevent degradation during processing - Processing under a nitrogen atmosphere is found to be more effective in preventing degradation than drying it prior to processing
Carrasco et al. [43]	PLA 2002D ^1^	SSE IM	Processing technique (IM, SSE + IM)	- IM caused only a slight decrease in molecular weight and a small increase in MFI, while IM + SSE showed a significant increase in the MFI and a significant decrease in molecular weight - PLA after SSE + IM shows a yellowish color due to degradation reactions, confirming the MFI and molecular weight results
Wang et al. [47]	PLLA ^4^	TSE	Temperature (180, 200 °C) Screw rotation speed (20, 50, 100, 200, 300, 400 rpm) Throughput (0.2, 0.34, 0.61 kg/h)	- Decreasing the throughput causes more degradation, due to a longer residence time and additional shear heating - Increasing the screw rotation speed results in a larger decrease in molecular weight due to more mechanical load on the polymer chains - Increasing the temperature in the barrel results in more thermal degradation
Cifuentes et al. [67]	PLLA ^5^ PLDA 2002D ^1^	TSE IM	Processing technique (TSE, IM)	- IM of PLDA causes a reduction of the Mv of 8%, where TSE of PLLA causes a reduction of 6%, both considered as negligible - Melting enthalpy of IM-PLLA is higher than values of TSE-PLDA, since injected material results in a higher crystalline fraction due to higher chain mobility given its shorter molecular weight - Processed materials have a higher crystallization rate than virgin PLA, due to degradation of the processed materials that causes a reduction in Mw and enhances crystallization
Mysiukiewicz et al. [79]	PLA 2500HP ^1^ PLA 3100D ^1^ PLA 3260D ^1^ PLA 4032D ^1^	TSE	Temperature (180, 200, 220, 240, 260 °C) Screw rotation speed (50, 100, 150, 200, 250 rpm) Initial MFI (7, 8, 24, 65 g/10 min)	- TSE at a high temperature and a low screw rotation speed results in a decreased viscosity, yellowing, and a reduction of molecular weight - High temperatures cause thermal degradation, while a low screw rotation speed elongates the residence time inside the extruder - Grades with a low initial molecular weight (high MFI) are less susceptible to degradation during TSE explained by a shorter residence time
Atalay et al. [86]	PLA 4060D ^1^ PLA 2500HP ^1^	TSE	D-isomer content (aPLA (12%), cPLA (0.5%)) Temperature (150, 190, 210, 230, 250 °C) Screw rotation speed (50, 100 rpm) Moisture content (25, 30, 60% RH at 60 °C)	- D-isomer content does not influence the thermal degradation behavior - Degradation of aPLA is negligible below 190 °C, but the degradation rate strongly increases for aPLA and cPLA beyond 190 °C - Processing at a lower screw rotation speed causes a larger decrease in complex viscosity due to a longer residence time - Conditioning at 25% and 30% RH shows similar degradation behaviors for aPLA and no effect for cPLA, but conditioning at 60% RH caused stronger degradation due to severe hydrolytic degradation for both aPLA and cPLA
Khankrua et al. [87]	PLA 2003D ^1^	TSE	Temperature (220, 230, 240, 250 °C)	- Molecular weight of processed PLA decreases with increasing temperatures due to severe thermal degradation reactions - Similar trends in MFI (increase in temperature causes increase in MFI) confirm the thermal degradation conclusions
Le Marec et al. [88]	PLA 7000D ^1^	CM LIM	Temperature (170, 190, 210 °C) Mixing time (10, 20, 30 min) Screw rotation speed (0, 40, 75, 150 rpm) Drying process (dried, undried)	- Degradation mainly depends on mixing time and temperature, since a shorter residence time and a lower temperature cause a smaller decrease in molecular weight - Increasing the screw rotation speed causes additional heating due to shearing of the screw and thus a larger decrease in the molecular weight - Drying PLA decreases the degradation rate constant with a factor 2
Taubner and Shishoo [89]	PLLA ^6^	LIM	Temperature (210, 240 °C) Screw rotation speed (20, 120 rpm) Moisture content (0%, 65% RH)	- Processing must be at a low temperature to minimize degradation, since the loss in molecular weight is less dependent on the residence time at the low temperature than at the high temperature - Moisture in PLA largely affects the molecular weight loss at the low temperature of 210 °C, but at the highest temperature of 240 °C the moisture is not contributing further to the degradation - The lowest speed rotation of 20 rpm elongates the residence time of the melt in the extruder and causes more degradation
Aldhafeeri et al. [90]	PLA 2003D ^1^	TSE QSE	Processing technique (TSE, QSE) Screw rotation speed (400, 1000 rpm) Screw configuration (less, more demanding) Throughput (2, 3, 4 kg/h)	- More complex melt flow in QSE causes a longer residence time inside the extruder and thereby more degradation when compared to TSE - A more demanding screw configuration (use of kneading blocks) and a lower screw speed elongate the residence time and thereby cause more degradation - Increasing the feed rate during QSE reduces the residence time and causes less degradation
Paakinaho et al. [91]	Medical grades ^7^	MSE	Initial inherent viscosity (2.18, 4.80, 6.26 dL/g)	- Higher initial molecular weight results in more molecular degradation - High molecular weight PLA-grades are affected by shear stress in the first third of the extruder and continue degrading until the melt viscosity was low enough to halt the degradation caused by the shearing action of the screw - Low molecular weight PLA is not affected by the extrusion process
Pantani et al. [92]	PLA 2002D ^1^	SSE IM	Processing technique (SSE, IM)	- SSE causes no significant degradation of PLA due to a short residence time of 1 min - IM causes a significantly lower viscosity when compared to virgin and extruded PLA due to a longer residence time of 15 min
Scoponi et al. [93]	PLA 4043D ^1^	TSE IM CM	Processing technique (TSE, IM, CM)	- TSE causes more degradation compared to IM, although an additional CM step after TSE deteriorates the material even further - Longer residence time during TSE and higher shear stresses compared to IM explain the results, since the material is longer exposed to high temperatures and shear stresses
Scaffaro et al. [94]	PLA 2002D ^1^	TSE TSC SSE	Processing technique (TSE, TSC, SSE)	- Processing PLA with TSE results in the highest viscosity and the lowest molecular weight decrease (less degradation), while TSC showed the lowest viscosity corresponding with a higher molecular weight decrease (more degradation) - Different processing techniques cause different residence times (shortest for TSE, longest for TSC) and different shear stresses

^1^ NatureWorks, ^2^ Total Corbion, ^3^ Cargill, ^4^ Biomer, ^5^ Total Corbion, ^6^ Neste Oy, ^7^ Purac Biochem.

## Data Availability

Data sharing not applicable.
